# Mechanically induced localisation of SECONDARY WALL INTERACTING bZIP is associated with thigmomorphogenic and secondary cell wall gene expression

**DOI:** 10.1017/qpb.2024.5

**Published:** 2024-05-03

**Authors:** Joshua H. Coomey, Kirk J.-M. MacKinnon, Ian W. McCahill, Bahman Khahani, Pubudu P. Handakumbura, Gina M. Trabucco, Jessica Mazzola, Nicole A. Leblanc, Rithany Kheam, Miriam Hernandez-Romero, Kerrie Barry, Lifeng Liu, Ji E. Lee, John P. Vogel, Ronan C. O’Malley, James J. Chambers, Samuel P. Hazen

**Affiliations:** 1 Biology Department, University of Massachusetts, Amherst, MA, USA; 2 Plant Biology Graduate Program, University of Massachusetts, Amherst, MA, USA; 3 Molecular and Cellular Biology Graduate Program, University of Massachusetts, Amherst, MA, USA; 4 US Department of Energy Joint Genome Institute, Lawrence Berkeley National Laboratory, Berkeley, CA, USA; 5 Institute for Applied Life Science, University of Massachusetts, Amherst, MA, USA

**Keywords:** gene expression, secondary cell wall, thigmomorphogenesis

## Abstract

Plant growth requires the integration of internal and external cues, perceived and transduced into a developmental programme of cell division, elongation and wall thickening. Mechanical forces contribute to this regulation, and thigmomorphogenesis typically includes reducing stem height, increasing stem diameter, and a canonical transcriptomic response. We present data on a bZIP transcription factor involved in this process in grasses. *Brachypodium distachyon* SECONDARY WALL INTERACTING bZIP (SWIZ) protein translocated into the nucleus following mechanostimulation. Classical touch-responsive genes were upregulated in *B. distachyon* roots following touch, including significant induction of the glycoside hydrolase 17 family, which may be unique to grass thigmomorphogenesis. SWIZ protein binding to an E-box variant in exons and introns was associated with immediate activation followed by repression of gene expression. *SWIZ* overexpression resulted in plants with reduced stem and root elongation. These data further define plant touch-responsive transcriptomics and physiology, offering insights into grass mechanotranduction dynamics.

## Graphical abstract

**Figure:**
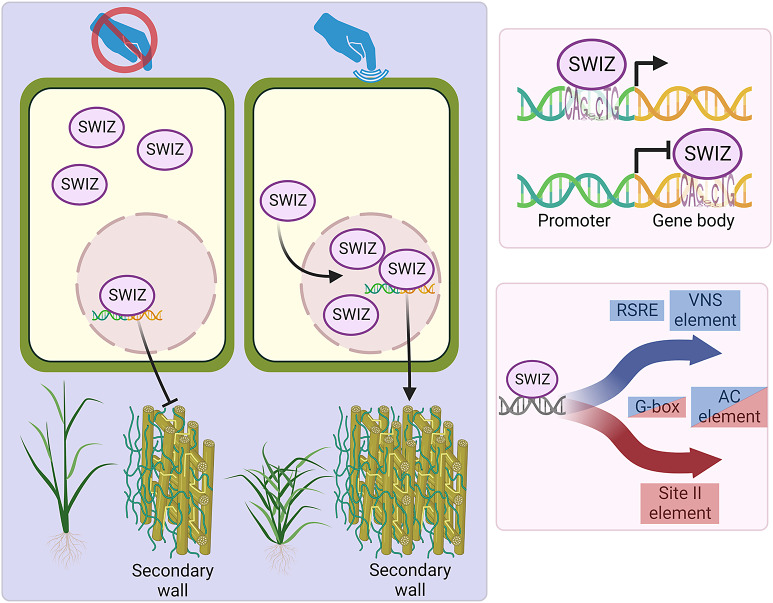

## 1. Introduction

Forces both internal and external to a cell - influence growth. Turgor pressure in conjunction with anisotropic cell wall dynamics directs plant cell shape and expansion. Force perception between neighbouring cells is critical in the development and maintenance of tissue form and function, such as the interlocking pavement cells on the leaf epidermis, or the developmental hotspots in the apical meristem (Bidhendi et al., 2019; Hamant et al., 2008; Uyttewaal et al., 2012). Specific inter-cell forces result in dynamic remodelling of the cortical cytoskeleton, with subsequent changes in cellulose microfibril alignment and alterations to other cell wall components such as pectin methyl esterification (Altartouri et al., 2019; Bidhendi et al., 2019; Bidhendi & Geitmann, 2018; Hamant et al., 2008; Uyttewaal et al., 2012). The classic hallmarks of touch-responsive growth, or thigmomorphogenesis, include reduced plant height, increased radial growth in plants with a cambial meristem, increased branching and delayed flowering time (Biro et al., 1980; Braam, 2004; Jaffe, 1973; Jaffe et al., 1980). These attributes have been leveraged by farmers for hundreds of years. As early as 1680, records show Japanese farmers tread on young wheat and barley seedlings to elicit increased branching, spikes per plant and grain weight per plant, along with stronger roots (Iida, 2014). This practice, known as mugifumi, continues today with mechanised rollers. Thigmomorphogenesis in belowground tissues has also been studied to some extent, with the impact of stiffer substrates eliciting changes in root length and straightness, with the implication of hormonal signalling pathways in mediating this response (Lee et al., 2019; Lourenço et al., 2015; Nam et al., 2020).

Mechanical stimulus can significantly remodel gene expression (Braam, 2004; Braam & Davis, 1990; Lee et al., 2005). The so-called *TOUCH* (*TCH*) genes in *Arabidopsis thaliana*, encode calmodulin (*AtTCH1*/*AtCaM2*), calmodulin-like proteins (*AtTCH2*/*AtCML24*, *AtTCH3*/*CML12*) and a xyloglucan endotransglucosylase/hydrolase (*AtTCH4*/*AtXTH22*) (Braam & Davis, 1990). Touch-responsive gene expression patterns often overlap with other stimuli such as dark, cold and hormone treatment (Lee et al., 2005; Polisensky & Braam, 1996). In addition to calcium binding and signalling, genes related to cell wall modification and a variety of transcription factors and kinases are regulated by mechanical stimulus, as well as genes involved in hormone homeostasis and signalling.

Group I bZIPs are also implicated in mechanosensing. VIRE2-INTERACTING PROTEIN 1 (AtVIP1) and related Group I bZIP proteins translocate from the cytoplasm to the nucleus in response to a variety of biotic and abiotic stimuli, including hypo-osmotic conditions (Tsugama et al., 2012, 2014, 2016). The Group I bZIP *Nt REPRESSOR OF SHOOT GROWTH* (*NtRSG*) in tobacco plays a role in maintaining GA homeostasis, wherein it translocates to the nucleus in response to cellular bioactive GA levels (Fukazawa et al., 2010; Igarashi et al., 2001; Ishida et al., 2008; Ito et al., 2017). Translocation appears to be dependent on protein phosphorylation, either from MITOGEN ACTIVATED PROTEIN KINASE 3 during pathogen invasion, or via calcium-dependent protein kinases. When phosphorylated, Group I bZIPs associate with 14–3–3 proteins in the cytoplasm until phosphatase activity releases them for nuclear translocation (Ishida et al., 2004; Ito et al., 2014, 2017; Tsugama et al., 2018; Van Leene et al., 2016).

Secondary cell walls deposited between the plasma membrane and primary cell wall provide mechanical strength in vascular and structural tissues. Secondary walls are made of crystalline cellulose, hemicelluloses and phenolic lignin polymers. Although functionally similar, secondary walls in monocotyledonous plants have key differences from eudicots, including distinct hemicellulose chemistry and differences in lignin biosynthesis. Grasses also produce mixed-linkage glucans (MLGs), a wall polysaccharide that is rarely found outside the commelinid monocots (Coomey et al., 2020). A tightly controlled network of feed-forward loops regulates the transcription of wall synthesising enzymes, with NAC family transcription factors activating wall synthesis genes as well as MYB family and other transcription factors that further promote secondary wall synthesis (McCahill & Hazen, 2019). These networks are similar between grasses and eudicots, with some components in each that have yet to be described in the other (Coomey et al., 2020). There are currently no bZIP family members in any secondary wall regulatory model.

Grasses employ a fundamentally different growth mechanism than eudicots, as they lack a cambium layer, and thus no lateral meristem. Stem elongation comes from division and elongation in a discrete series of intercalary meristems, called nodes, with one internode region elongating and pushing up subsequent nodes. Detailed studies of thigmomorphogenesis have been conducted almost exclusively in eudicots. However, recent work in the model cereal grass *Brachypodium distachyon* shows general overlap with conventional eudicot thigmomorphogenesis but with no change in stem diameter and increased time to flower (Gladala-Kostarz et al., 2020).

Thigmomorphogenesis is a widely observed phenomenon that results in reduced height, increased radial growth and increased branching. The mechanisms behind this form of growth are not yet fully understood, but involve aspects of hormone regulation, Ca^2+^ signalling, Group I bZIP intracellular translocation and changes in gene expression. Here we describe the transcriptional response to mechanical stimulation and the function of a *B. distachyon* bZIP transcription factor, SECONDARY WALL ASSOCIATED bZIP (Bradi1g17700) and its role in touch response and cell wall biosynthesis.

## 2. Results

### 2.1. SWIZ is a Group I bZIP transcription factor and candidate cell wall regulator

To identify genes involved in the regulation of secondary cell wall thickening, Trabucco et al. (2013) measured transcript abundance in *B. distachyon* leaf, root and stem tissue. A gene annotated as a bZIP transcription factor, Bradi1g17700, was highly expressed in root and stem relative to leaf (Supplemental Figure S1A). Bradi1g17700 is also a member of a 112-gene coexpression network (Supplemental Table S1) that includes genes highly expressed in the peduncle (Sibout et al., 2017). Phylogenetic analysis of Bradi1g17700, hereinafter referred to as SECONDARY WALL INTERACTING bZIP (SWIZ), amino acid sequence shows it to be an ortholog of the *A. thaliana* Group I bZIPs (Dröge-Laser et al., 2018; Jakoby et al., 2002) and closely related to *AtbZIP18* and *AtbZIP52* (Supplemental Figure S1B, Supplemental File S1).

### 2.2. SWIZ translocates into the nucleus in response to mechanical stimulus

**Figure 1.**
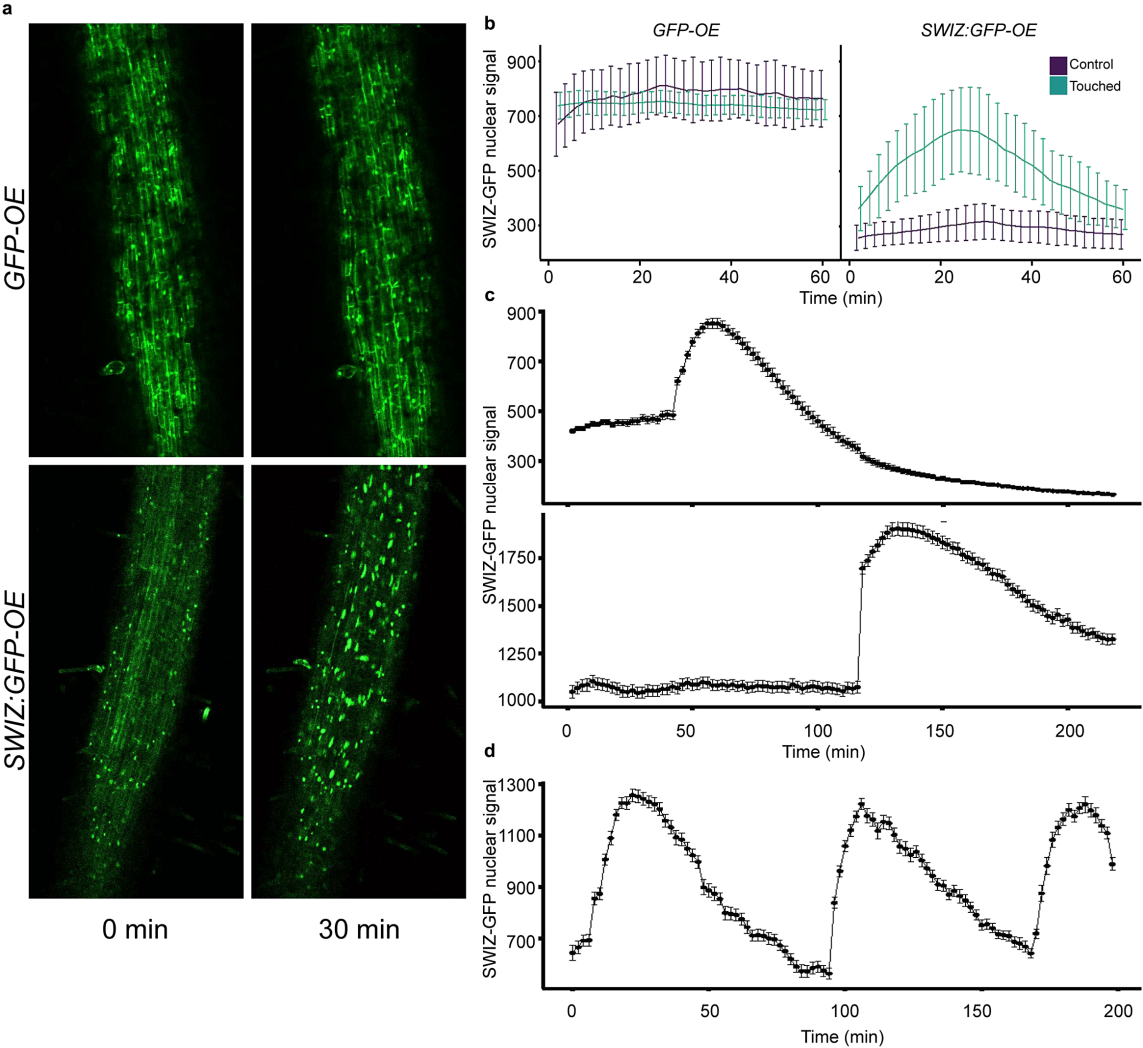
SWIZ translocates to the nucleus in response to mechanical stimulus, specifically in regions directly stimulated. (a) Image of *SWIZ:GFP-OE* and *GFP-OE* roots prior to stimulus and 30 min post-stimulus. Roots were observed immediately following mechanical perturbation. (b) Quantification of nuclear signal in control (purple) and touched (teal) conditions for *GFP-OE* (left) and *SWIZ:GFP-OE* (right). *n* = 14–20 nuclei. (c) SWIZ translocation occurred in the local area of the stimulus. At 30 min, stimulus was applied to an upper region of the root, while at 120 min it was applied to a lower region approximately 3 cm below. *n* = 109 and 184 nuclei, respectively, for upper and lower regions. Scale bar = 100 μm, (d) *SWIZ:GFP-OE* roots were imaged by confocal microscopy with stimulus applied in the field of view at 0, 90 and 180 min. *n* = 126 nuclei. (b–d) Images were taken every 2 min. Nuclear GFP signal was quantified in selected nuclei at each time point. The average nuclear GFP signal is represented by the line with error bars indicating the standard error of the mean. Scale bar = 100 μm. *n* = 4–6 plants per treatment.

Group I bZIPs in *A. thaliana* translocate between the cytosol and nucleus in response to external cellular force. As an ortholog of these proteins, we hypothesised that SWIZ protein may similarly translocate within the cell in response to mechanical force. To test this, the roots of plants overexpressing either *SWIZ* fused to *GFP* (*SWIZ:GFP-OE*) or *GFP* alone (*GFP-OE*) were observed following a mechanical stimulus (Figure 1a, Supplemental Files 2 and 3). In control plants, GFP signal was both cytosolic and nuclear, which remained constant over the imaging period (Figure 1b). GFP signal was mostly observed in the cytosol in *SWIZ:GFP-OE* plants, but following mechanical stimulus, nuclear GFP signal increased substantially, peaking around 30 min post-stimulus and returned to near basal levels by ~60 min, while untouched plants showed no change in signal localisation (Figure 1b).

After repeated stimulus in some systems, the plant touch response can become desensitised (Leblanc-Fournier et al., 2014; Martin et al., 2010; Moulia et al., 2015). To test if SWIZ translocation dynamics varied after repeated treatments, we applied mechanical force to *SWIZ:GFP-OE* roots at regular intervals. A second stimulus was given 90 min after the first, and a third at 180 min. Following each mechanical stimulation, SWIZ consistently translocated from cytoplasm to the nucleus (Figure 1d). This suggests that SWIZ translocation dynamics are not impacted by repeated stimulus events 90 min apart.

To determine if the signal triggering SWIZ translocation is spread beyond the specifically stimulated region, two regions of the same *SWIZ:GFP-OE* root separated by 3 cm were simultaneously observed. The stimulated region showed typical SWIZ:GFP nuclear signal accumulation and in the region below no translocation was observed (Figure 1c). At 120 min, the treatments were reversed, with the lower root region receiving a stimulus while the upper region was unperturbed. The lower region showed SWIZ:GFP nuclear translocation while the upper region did not. Thus, SWIZ touch-mediated translocation is a local response at the mechanically stimulated region.

### 2.3. Root transcriptional response to touch

**Figure 2.**
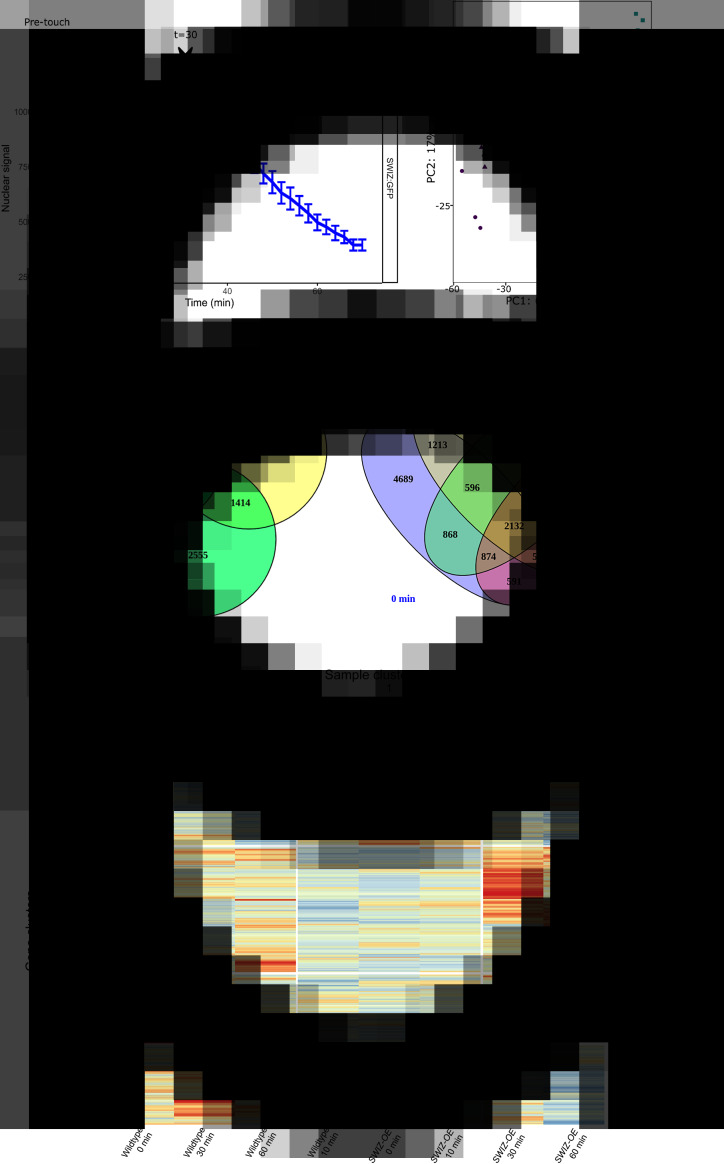
Transcriptome analysis of touch response in *Brachypodium distachyon* roots. (a) Root tissue was sampled just prior to touch (*t* = 0), and at 10, 30 and 60 min following touch treatment in wildtype and *SWIZ-OE.* (b) Principal component analysis of gene expression across samples shows the greatest difference corresponding to genotype and the second greatest corresponding to time after touch. (c) Venn diagram analysis of unique and overlapping differentially expressed genes in wildtype at 10, 30 and 60 min following touch treatment, relative to *t* = 0 min. (d) Venn diagram analysis of unique and overlapping differentially expressed genes in *SWIZ-O*E at 0, 10, 30 and 60 min following touch treatment, relative to wildtype at *t* = 0 min. (e) Heatmap depiction of hierarchical clustering of gene expression direction and magnitude in wildtype and *SWIZ-OE* at *t* = 0, 10, 30 and 60 min following touch treatment. Genes with zero-expression in any sample were excluded. Euclidean distance with complete clustering was used to generate the hierarchy, with k-means grouping to organise the boxes.

Having established the nuclear translocation of SWIZ in response to touch, we then investigated what effect touch and *SWIZ* overexpression during touch may have on gene expression. We measured transcript abundance by sequencing mRNA from wildtype and *SWIZ-OE* root tissue just before touch (0 min) and at 10, 30 and 60 min following touch treatment along the entire length of the root (Figure 2a, Supplemental Figure S2). Principal component analysis of transcript abundance shows the greatest amount of variance is attributed to the first principal component, 60%, where samples cluster based on genotype (Figure 2b). The second principal component, which accounts for 17% of the variance, distinguished between pre-touch and 10 min following touch, with the last two time points clustering similarly.

The wildtype transcriptome underwent significant remodelling in response to touch, with 8,902 transcripts differentially expressed (*q* < 0.1) at 10 min post-touch, 5,682 transcripts at 30 min and 7,672 transcripts at 60 min (Supplemental Tables S2–S4). *SWIZ* itself was strongly upregulated following touch in *SWIZ-OE* (Supplemental Figure S2). Before touch treatment, *SWIZ-OE* exhibited 11,488 differentially expressed genes compared to untouched wildtype roots. Furthermore, the *SWIZ-OE* transcriptome was substantially altered by touch relative to wildtype, with 5,757 transcripts differentially expressed at 10 min post-touch, 6,330 transcripts at 30 min and 5,320 transcripts at 60 min. The overlap between these groups is depicted in Figure 2c,d. Additionally, we compared differentially expressed genes in wildtype and *SWIZ-OE* both before and after touch (Figure 2e). Hierarchical clustering was employed to visualise the similarity between genotypes and time course samples. Genes in cluster 1 were generally upregulated in wildtype and downregulated in *SWIZ-OE*. Notably, wildtype at 10 min post-touch exhibited the greatest similarity to pre-touch *SWIZ-OE* and 10 min post-touch *SWIZ-OE*, indicating that the untreated *SWIZ-OE* displayed gene expression patterns akin to the early-touch response in wildtype. These three samples were similarly and uniquely downregulated in gene clusters 2 and 3, while gene cluster 4 was upregulated. These shifts correlated with the timing of SWIZ protein accumulation in the nucleus following touch.

**Figure 3.**
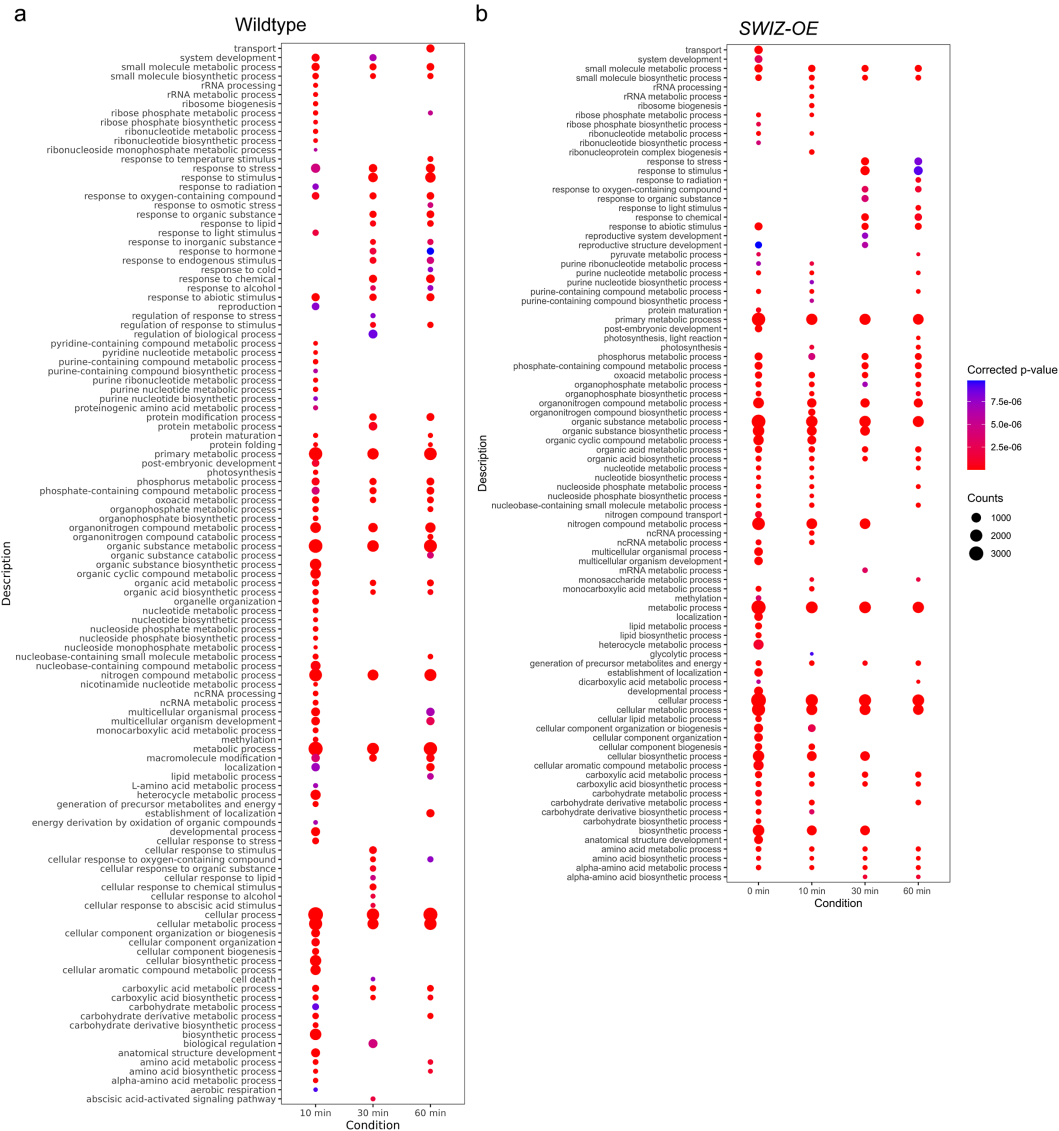
Gene ontology (GO) analysis of differentially expressed genes in wildtype and *SWIZ-OE* following touch treatment. (a) GO terms for biological processes for differentially expressed genes in (a) wildtype and (b) *SWIZ-OE* samples following touch. Dot colour indicates corrected *p*-value, while the dot size indicates the number of read counts.

To further investigate the transcriptomic responses of wildtype and *SWIZ-OE*, we conducted gene ontology (GO) analysis for biological processes for the differentially expressed transcripts at each time point (Figure 3). Following touch, numerous terms were enriched in wildtype samples, encompassing various cellular processes including response to stimulus, stress, abiotic, osmotic and cold (Figure 3a). Interestingly, untouched *SWIZ-OE* exhibited a strikingly similar profile to wildtype at 10 min, mirroring the findings of hierarchical clustering (Figure 2d). While the GO terms in *SWIZ-OE* were similar to those enriched in wildtype samples, certain distinctions emerged. For instance, terms related to response to stress and stimulus appeared later in wildtype, specifically at 30 and 60 min post-touch. Additionally, terms associated with metabolic and biosynthetic processes showed greater persistence in *SWIZ-OE* across most time points, contrasting with their appearance mainly at 10 min post-touch in wildtype, with more variability at 30 and 60 min (Figure 3b).

**Figure 4.**
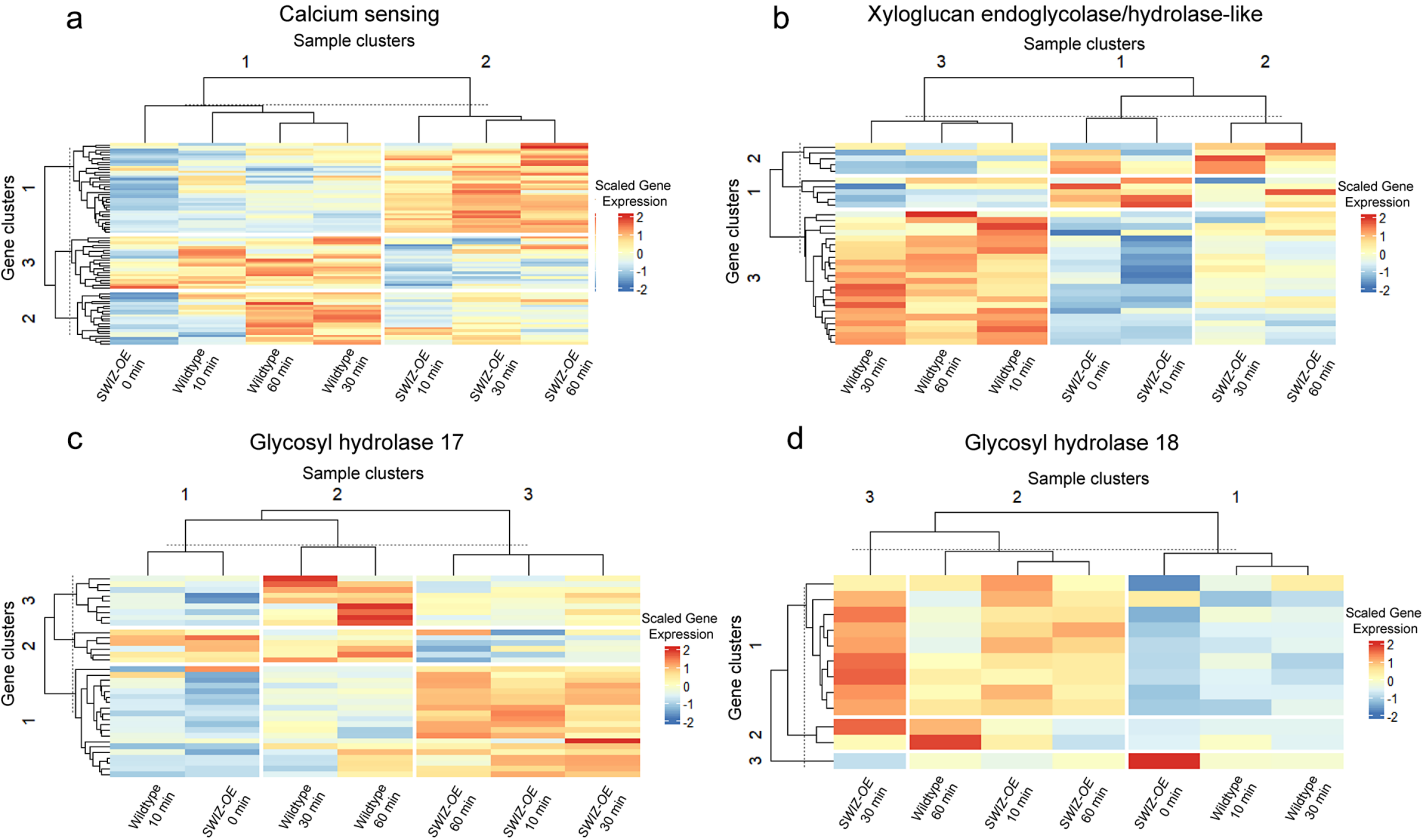
Gene expression patterns of canonical and novel touch-responsive genes in wildtype *B. distachyon* and *SWIZ-OE* following touch.Differential gene expression of (a) calcium binding (b) xyloglucan endoglycolase/hydrolase (XTH), (c) glycosyl hydrolase 17 (GH17) and (d) GH18 family for wildtype and *SWIZ-OE* at 0, 10, 30 and 60 min following touch treatment. Full list of genes for calcium binding, XTH and glycoside hydrolases defined by Tyler et al. (2010) is provided in Supplemental Tables S11–S13. Heatmaps were made with expression valves scaled to the median of three RNA-seq replicates with Euclidean distance and complete clustering was used to generate the hierarchy and k-means grouping to organise the boxes.

Canonical touch-responsive genes include calcium-sensing proteins such as CAMs, CmL and XTHs (Braam & Davis, 1990; Lee et al., 2005). Based on homology with *TCH1*, *TCH2* and *TCH3*, we identified 79 calcium sensing genes, the majority of which exhibited upregulation in wildtype plants following touch treatment (Figure 4a, Supplemental Table S11). Notably, *SWIZ-OE* plants also showed upregulation of calcium-sensing genes, albeit with a distinct subset responding over time. *SWIZ-OE* pre-touch closely resembled wildtype plants at 10 min post-touch, as seen in the hierarchical clustering and GO term analysis (Figures 2d, 3, 4a). However, at 30 and 60 min, gene clades 2 and 3 showed upregulation in wildtype plants post-touch. In contrast, gene clade 1 displayed consistent upregulation in *SWIZ-OE* across 10, 30 and 60 min, while remaining relatively stable in wildtype plants (Figure 4a, Supplemental Table 11).

**Figure 5.**
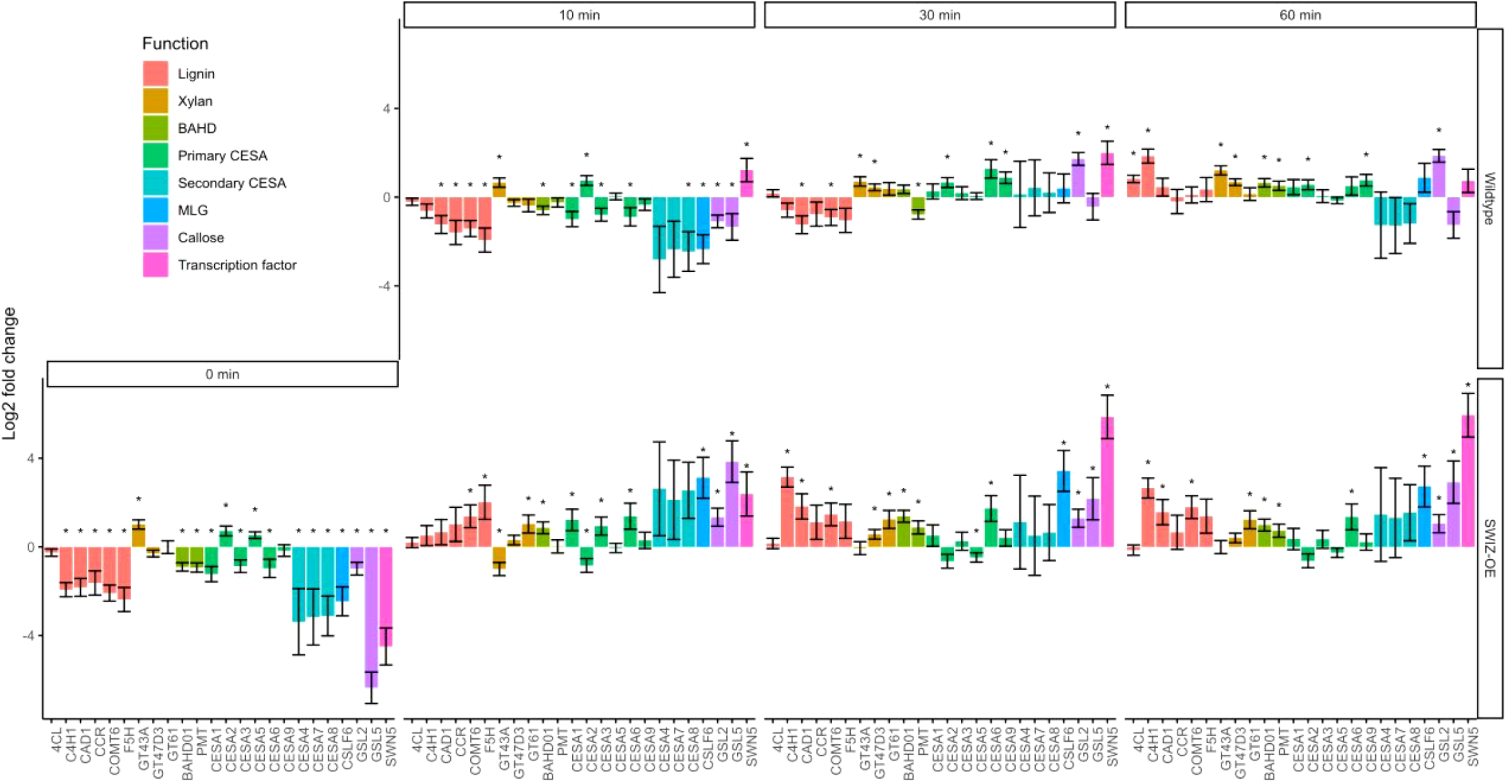
Gene expression analysis of cell wall-related genes. Log fold-change of gene expression measured by RNA-seq in wildtype and *SWIZ-OE*, presented as relative to wildtype expression at time 0, pre-touch. Bar colour indicates class of cell wall gene. Error bars indicate standard deviation of three biological replicates. Significance denoted by * reflecting *q* < 0.1 compared to wildtype expression at *t* = 0, with *q*-values representing Wald test *p*-values adjusted for false discovery rate. Legend abbreviations: BAHD, BAHD (BEAT, AHCT, HCBT and DAT) acyltransferases; CESA, cellulose synthase; MLG, mixed-linkage glucans.

Additionally, the 37 *B. distachyon* XTH and XTH-like genes, resembling *TCH4*, were expressed in both *SWIZ-OE* and wildtype plants at 10 min post-touch (Figure 4b, Supplemental Table S12). This clade, along with sample clade 2, exhibited upregulation of a subset of XTH genes (gene clades 1 and 2) in both *SWIZ-OE* and wildtype plants across all time points. However, this group of genes experienced notable downregulation in *SWIZ-OE* at 10 and 30 min, with a subsequent increase observed at 60 min. Moreover, *SWIZ-OE* diverged from wildtype plants in gene clade 3, exhibiting modest upregulation in wildtype at 30 min but pronounced upregulation in *SWIZ-OE* at 10, 30 and 60 min.

Cell wall modification is closely linked to touch-responsive growth, evident in both the gene expression patterns of XTH genes and observable changes in plant stature (Gladala-Kostarz et al., 2020; Lee et al., 2005). Given this association, we investigated the response of glycosyl hydrolase genes in wildtype and *SWIZ-OE* plants following touch stimulation. This gene superfamily plays diverse roles in modifying various wall polymers. Our study unveiled a previously unreported observation: the significant upregulation of glycosyl hydrolase family 17 (GH17) and GH18 families following touch. Specifically, the GH17 family (Tyler et al., 2010), known for modifying β-1,3-glucans, exhibited notable changes. Among the 53 annotated GH17 members, 38 were measured. In wildtype plants, 8, 11 and 16 members showed differential expression at 10, 30 and 60 min, respectively, while in *SWIZ-OE* plants, the numbers were 22, 7, 10 and 7 at 0, 10, 30 and 60 min, respectively. Fisher’s exact test revealed significant enrichment of GH17 expression at 60 min in wildtype (adjusted *p* = 0.04) and at 0 min in *SWIZ-OE* (adjusted *p* = 6.15 × 10^–3^) (Figure 4c, Supplemental Table S13). Notably, *SWIZ-OE* plants at 0 min exhibited a gene expression pattern like wildtype plants at 10 min. In the sample clade analysis, wildtype plants at 30 and 60 min clustered together in sample clade 2, with pronounced upregulation of some GH17 members in gene clades 2 and 3, and a mix of downregulation and modest upregulation in gene clade 1. Conversely, *SWIZ-OE* plants at 10, 30 and 60 min grouped in sample clade 3, showing strong upregulation of gene clade 1, and downregulation mixed with modest upregulation of genes in gene clades 2 and 3.

The GH18 family was overrepresented with differentially expressed genes in just *SWIZ-OE* at 30 min following touch (Figure 4d). GH18 enzymes are characterised as chitinases, and are involved in modifying and hydrolysing chitin oligomers, typically in fungal cell walls. Fisher’s exact test determined significant enrichment of GH18 expression at 30 min (adjusted *p* = 7.53 × 10^–3^). The pattern of expression of this family shows overall repression in *SWIZ-OE* at 0 min, similar to wildtype at 10 and 30 min. *SWIZ-OE* showed general upregulation at 10, 30 and 60 min, as does wildtype at 60 min.

Broadly speaking, *SWIZ-OE* expression levels at 0 min closely resemble those of the wildtype but diverged notably at subsequent time points. *SWIZ-OE* exhibited a pronounced upregulation of genes that were modestly induced in the wildtype, alongside an inverse regulation pattern for certain subsets of genes across various time points.

An interactive platform for exploring this *B. distachyon* touched-root transcriptome further is available online at (https://hazenlab.shinyapps.io/swiztc/).

### 2.4. *Specific cell wall genes are downregulated immediately following touch, then induced concurrent with SWIZ nuclear translocation and more strongly in* SWIZ-OE

Given the impact of touch on certain cell wall modifying genes as described above, and the nature of macro observations of plant stature following touch, we chose to explore specific cell wall gene expression patterns. In particular, we pursued secondary cell wall synthesis and the grass-specific MLG-related genes that have been characterised in the literature (Coomey et al., 2020). Genes related to secondary cell wall synthesis were immediately downregulated in wildtype plants following touch but were subsequently upregulated (Figure 4, Supplemental Table S5–S10). Xylan, cellulose, MLG, and callose synthesis-associated genes were upregulated at 30 and 60 min following touch, while lignin biosynthesis and BAHD acyltransferase genes were upregulated at 60 min. Lignin biosynthesis originates from the shikimate biosynthetic pathway, comprising several enzymatic steps to produce monolignols, which are subsequently radicalised to facilitate polymerisation. Genes encoding enzymes across all steps of this process were upregulated following touch, with strong induction in *SWIZ-OE* at 10, 30 and 60 min. Before touch, nearly all cell wall-associated genes investigated were significantly downregulated relative to wildtype, then significantly upregulated following touch (Figure 5). In *SWIZ-OE*, cell wall-related terms were enriched in paths that had stronger upregulation earlier than the same terms in wildtype (Supplemental Figure S3A-B). Network analysis of gene expression patterns over time using the iDREM software showed that in *SWIZ-OE* plants, cell wall genes were more rapidly induced. Notably, cell wall polysaccharide biosynthesis, particularly glucan biosynthesis, was significantly enriched in *SWIZ-OE* path D, while not significantly enriched in wildtype (Supplemental Table S14). This is mirrored when looking at specific gene expression, with primary wall CESAs, MLG, and callose synthesis genes all significantly upregulated following touch in *SWIZ-OE*. Thus, cell wall genes were immediately repressed by touch, followed by significant activation, which occurs more rapidly in *SWIZ-OE* plants and is associated with the timing of SWIZ nuclear translocation.

**Figure 6.**
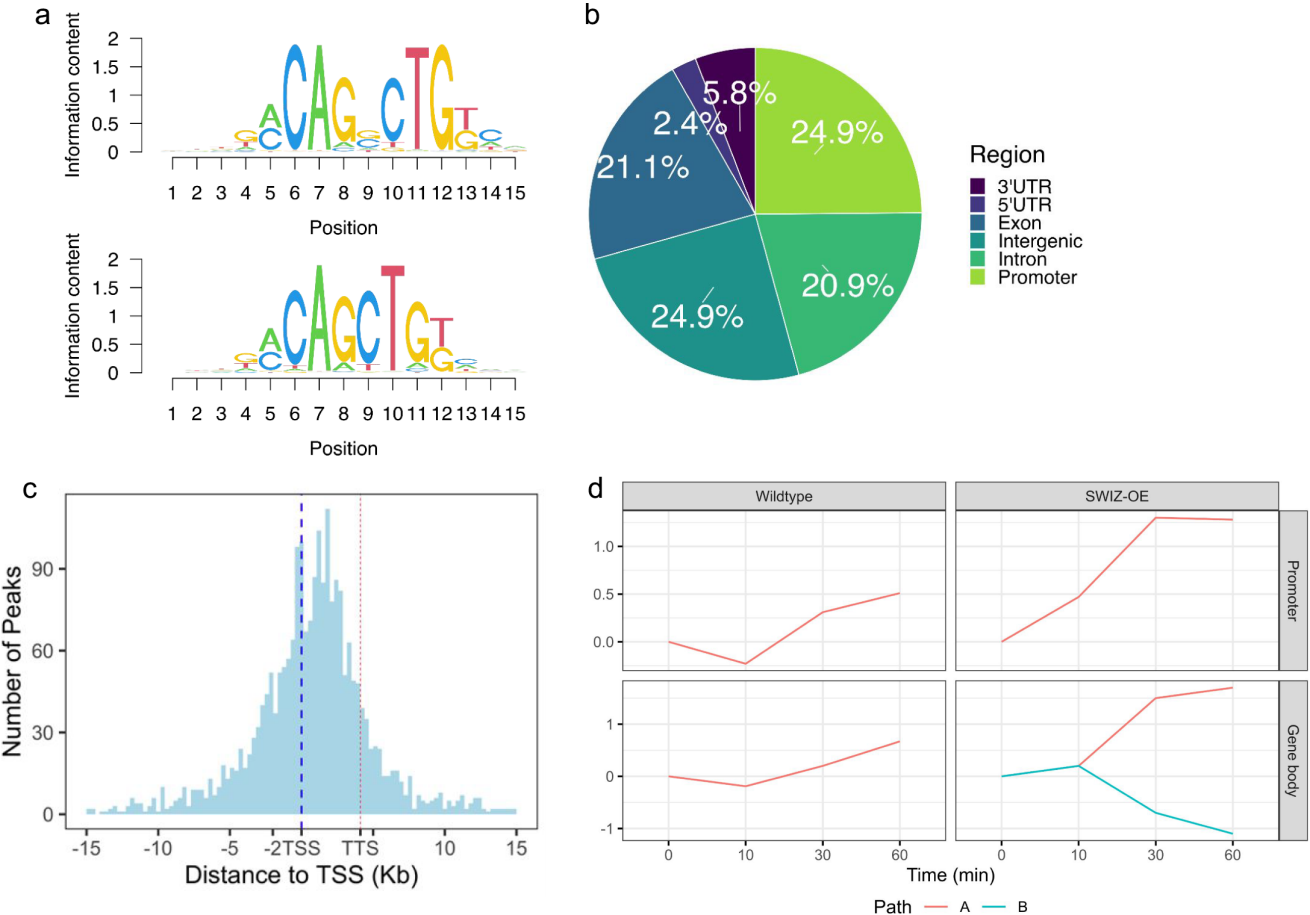
DNA affinity purification sequencing to determine SWIZ binding sites (a) Top two most statistically enriched sequence motifs in SWIZ binding sites. (b) Distribution of binding sites across genomic features, relative to primary transcripts of the *Brachypodium distachyon* annotation v 3.1. (c) Relative distribution of binding sites centred on the transcriptional start site (TSS, blue dashed line), transcriptional termination site (TTS, red dashed line) represents the average length of all annotated transcripts, approximately 4.5 kb away from the TSS. (d) Path determinations from iDREM time course analysis of differentially expressed genes that also have DAP-seq binding sites. Each line represents a set of genes with similar expression level patterns over the time course relative to time 0, pre-touch. Wildtype: promoter and gene body = 170 and 146 genes, respectively. *SWIZ-OE*: promoter = 163 genes, gene body A and B = 141 and 60, respectively.

### 2.5. SWIZ protein binding in the gene body is associated with dynamic changes in gene expression

Next, we investigated the direct binding targets of SWIZ protein by performing DNA affinity purification sequencing (DAP-seq) with whole genomic DNA. SWIZ interacted with 2,455 distinct positions in the genome (Supplemental Table S15). Those regions were significantly enriched for two E-box motif variants, (A/C)CAGNCTG(T/G) and (A/C)CAGCTG(T/G) (Figure 6a). Numerous binding sites were between the translational start and stop sites, with 21% of peaks in introns and 21% in exons (Figure 6b). Fewer than 10% of the peaks occurred in UTRs and the promoter (5 kb upstream of the 5‘UTR) and intergenic regions each accounted for 25%. Thus, SWIZ protein is preferentially bound to an E-box-like motif and often in the gene body (Figure 6c).

**Table 1:** Number of SWIZ DAP-seq targets differentially expressed in wildtype plants after touch treatment relative to untouched wildtype.

Time after touch	Promoter		Gene body	
	Up	Down	Up	Down
(min)	percent (count)			
0	42.0 (103)	58.0 (142)	48.0 (286)	52.0 (310)
10	57.0 (73)	43.0 (55)	53.0 (167)	47.0 (148)
30	59.4 (79)	40.6 (54)	41.2 (136)	58.8 (194)
60	64.2 (77)	35.8 (43)	45.7 (128)	54.3 (152)

Note: The location of SWIZ DAP-seq binding, in either the promoter or gene body regions, is noted as is the direction of differential expression, up or down.

We next compared genes differentially expressed in the *SWIZ-OE* touch response RNA-seq time courses with the binding targets identified by DAP-seq (Supplemental Tables S5–S9, S15 **)**. Before touch, genes with promoter-SWIZ interactions were most often downregulated in *SWIZ-OE* relative to wildtype (Table 1). Following touch, SWIZ promoter binding targets were most often upregulated in *SWIZ-OE* in all three post-touch time points. Thus, SWIZ promoter binding was coincident with repression, but following a touch stimulus, activation was more prominent. This difference between untreated and touched was not as pronounced in gene body targets, with more upregulated genes 10 min following touch and more downregulated 30 and 60 min following touch. To further explore these trends, we conducted an iDREM cluster analysis of the differentially expressed genes that were also SWIZ protein targets (Figure 6d). For both wildtype and *SWIZ-OE*, the network analysis revealed a pathway to increased gene expression by 60 min following touch. The network pathway trend for both promoter and gene binding targets in wildtype was immediate repression at 10 min followed by increased expression at 30 and 60 min post-stimulus. A unique pathway was observed among gene body binding targets in *SWIZ-OE*; transcript abundance was reduced following touch. Thus, SWIZ binding to a promoter or gene body was more strongly associated with increased expression except for gene body targets in touched *SWIZ-OE* plants, which were more strongly repressed.

**Figure 7.**
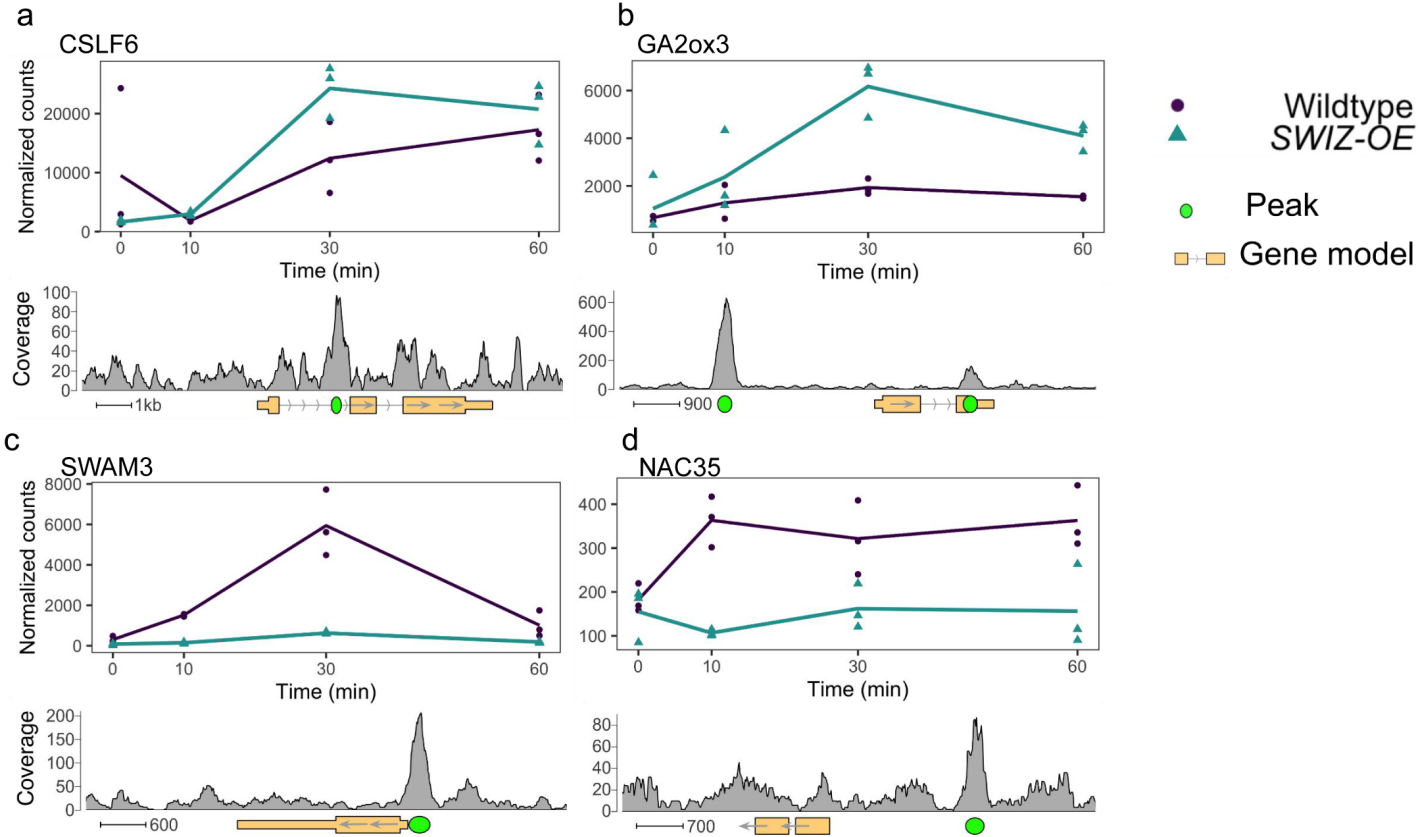
SWIZ binding targets differentially expressed in response to touch and *SWIZ-OE*. Gene expression over time of selected genes with SWIZ binding sites: (a) CSLF6, (b) GA2ox3, (c) SWAM3, and (d) NAC35. Line graphs are the average transcript abundance of three biological replicates for each time point. Binding site determined as peaks of sequence alignment. Scale bar unit is bases. Direction of transcription is shown with arrows on the gene model, 5’ and 3’ UTRs are depicted by narrowed rectangles on the gene model.

Several vignettes stood out as examples of genes differentially expressed by touch, *SWIZ-OE* transgene, and being bound by SWIZ at various locations. The mixed glucan synthase *CSLF6* was bound in the first intron and upregulated by touch and *SWIZ-OE* (Figure 7a). *GA2ox3*, which inactivates bioactive GA, was bound by SWIZ protein in both the gene body and 3’UTR and was upregulated 30 min after touch in *SWIZ-OE* (Figure 7b). An example of gene body binding repression is *SWAM3* (Bradi1g30252), the closest ortholog to a wheat transcription factor induced by hypoxia, *TaMYB1* (Handakumbura et al., 2018; Lee et al., 2006) (Figure 7c). A membrane-associated transcription factor, NAC35, was bound by SWIZ in the promoter region and downregulated in SWIZ-OE (Figure 7d).

### 2.6. Cis-regulatory sequences associated with mechanical stress, wounding and cell wall synthesis are enriched among touch-responsive genes

**Figure 8.**
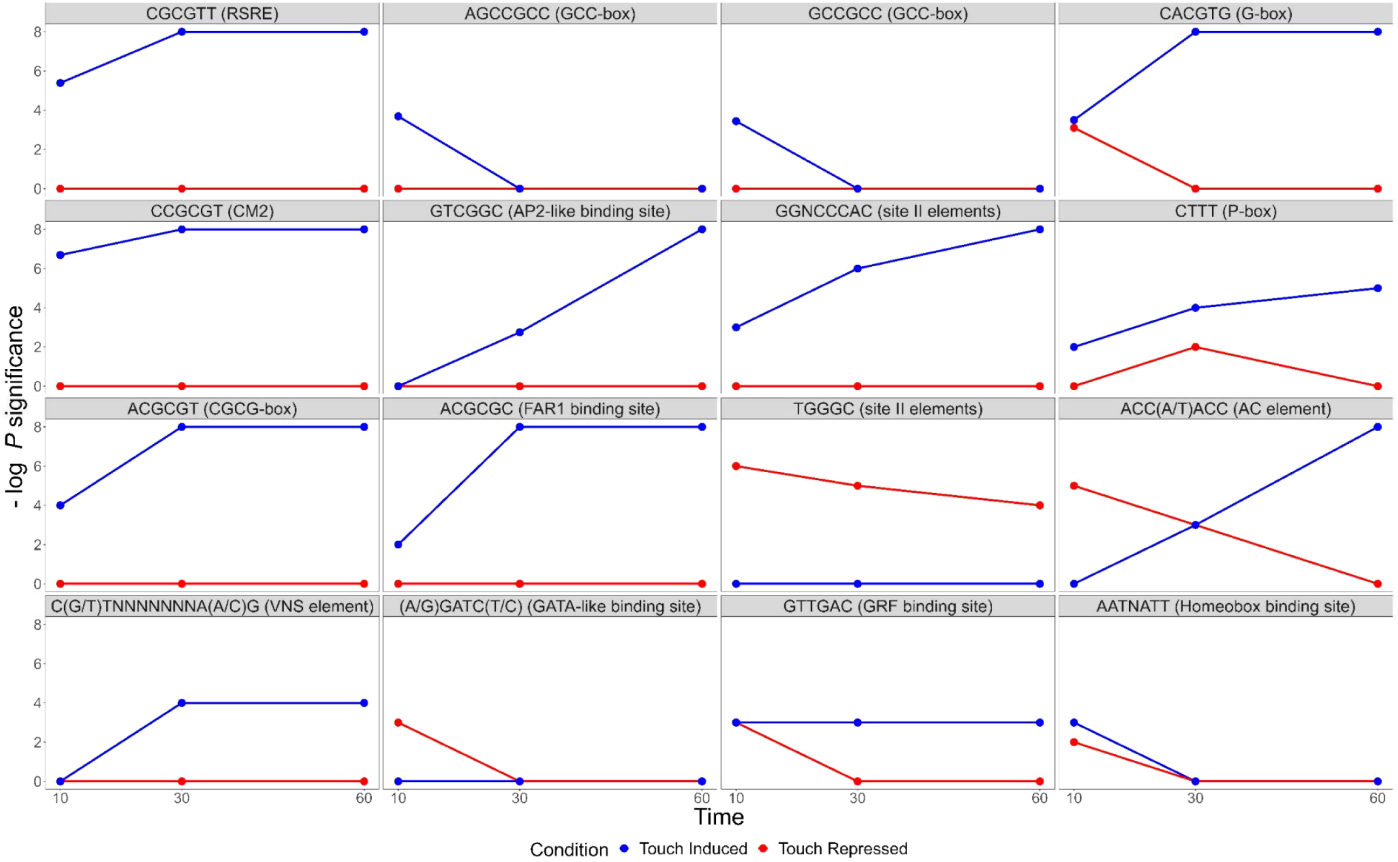
Sequence motifs enriched in the *cis*-regulatory regions of touch-responsive *Brachypodium distachyon* genes. Negative log *p-*values for *cis*-elements, known and not known to be touch-responsive. RSRE – rapid stress response element, FAR1 – FAR-RED impaired response1, GRF – growth-regulating factor, VNS – VND, NST/SND and SMB.

Touch-responsive genes in wildtype were analysed for enrichment of putative *cis*-regulatory elements (CREs). We identified several sequences significantly enriched among touch-responsive transcripts (Figure 8, Supplemental Figure S4, Supplemental Table S16, S17). Several of those have been previously described as touch-responsive, including the Rapid Stress Response Element, the GCC boxes AGCCGCC and GCCGCC, a sequence referred to as both the E-box and G-box (CACGTG), CM2, AP2-like, GGNCCCAC site II element, P-box and the GRF and FAR1 binding sites (Doherty et al., 2009; Fernández-Calvo et al., 2011; Moore et al., 2022; Rushton et al., 2002; Walley et al., 2007). Other putative CREs have not previously been identified as touch-responsive. One CRE was exclusively enriched in touch-repressed genes at all time points, the TCP site II element TGGGC. A GATA-like binding site was enriched among 10 min repressed transcripts. The homeobox binding site motif was enriched among both induced and repressed genes. The CGCG-box was enriched among induced genes at all timepoints like RSRE, CM2 and FAR1. The two CREs associated with secondary cell wall thickening were also significantly enriched (Coomey et al., 2020); the VNS element among induced genes, and the AC element ACC(A/T)ACC with a unique profile. AC elements were enriched among repressed genes at 10 min, in both induced and repressed genes at 30 min, and among induced genes only at 60 min.

### 2.7. Root morphology is altered by SWIZ-OE and mechanical treatment

Given the SWIZ protein mechanical response and regulatory influence, we then investigated how SWIZ might impact plant growth in response to touch. We challenged wildtype and *SWIZ-OE* roots with the same style of touch treatment used in the translocation and gene expression experiments but repeated twice daily for a period of 5 days. In both touch and control conditions, *SWIZ-OE* roots were significantly shorter than wildtype, suggesting a dwarfing effect from *SWIZ* overabundance (Supplemental Figure S5A-C). Mechanical challenges to roots have been reported to impact root straightness, a trait that has also been described as being impacted in other bZIP studies (Van Leene et al., 2016; Zha et al., 2016). In control conditions, we observed that *SWIZ-OE* roots were significantly less straight than wildtype, while this was not observed in response to touch (Supplemental Figure S5D). We further tested the mechanoresponse of *SWIZ-OE* roots by growing seedlings on plates with increasing degrees of plate angle at 10°, 20°, 30° and 40° from vertical as a form of mechanostimulation (Oliva & Dunand, 2007; Zha et al., 2016) (Supplemental Figure S6). Wildtype *B. distachyon* roots displayed decreasing root length with increasing plate angle. *SWIZ-OE* roots were shorter than wildtype at all plate angles tested (Supplemental Figure S6). Root straightness did not show any significant differences.

### 2.8 *B. distachyon* displays classic thigmomorphogenic phenotypes

We next investigated the effect of touch treatment on aboveground tissue. Wildtype plants were perturbed with a metal bar once every 90 min for either 2 or 3 weeks (Supplemental Figures S7 and S8). After the treatment period, all plants were allowed to recover and grow to senescence (Supplemental Figure S8). Two-week stressed plants were significantly shorter than control plants, and 3-week stressed plants were shorter still (Supplemental Figure S8A-B). Despite this difference in height, there was no significant difference in aboveground biomass (Supplemental Figure S8C **)**. Three-week stressed plants had significantly more branches, (Supplemental Figure S8D). Transverse stem cross sections in the third elongated internode and peduncle did not show a significant difference in cell wall thickness or phloroglucinol staining in response to touch (Supplemental Figure S8E-G).

### 2.9. Stem height and cell wall thickening are affected by SWIZ-OE and touch treatment

To test the role of *SWIZ* in regulating thigmomorphogenesis and secondary cell wall development, we tested wildtype and *SWIZ-OE* under 2 weeks of mechanical perturbation as described above. In control conditions, there were no differences among genotypes in height, weight, or branching. Touch significantly shortened both wildtype and *SWIZ-OE* stems relative to control conditions, but not relative to each other (Supplemental Figure S9, S10). Transverse sections of the stem were made in the peduncle, the last elongated stem internode where the touch treatment occurred during stem elongation, and stained with phloroglucinol-HCl. *SWIZ-OE* touched peduncles showed significantly thicker interfascicular fibre cell walls compared to untouched, but not significantly different from touched wildtype (Supplemental Figure S10B-C).

## 3. Discussion

Touch stimulus is generally an inhibitor of plant elongation growth, but promotes branching and radial expansion (Braam, 2004; Chehab et al., 2009; Jaffe, 1973). The majority of this work has been done in dicots, where increased radial growth has been associated with greater deposition of secondary wall-forming cells, particularly in woody species such as poplar (Biro et al., 1980; Börnke & Rocksch, 2018; Coutand et al., 2009; Niez et al., 2019; Roignant et al., 2018). Our understanding of thigmomorphogenesis in grasses is limited, and mostly in the agricultural context of lodging (Shah et al., 2019). While these studies highlight the importance of stem strength and associated cell wall defects, they do relatively little to elucidate the mechanosensitive response. Recent work by Gladala-Kostarz et al. (2020) describes grass touch response to both wind and direct mechanical treatment, with an emphasis on cell walls and stem anatomy. Touch treatment significantly increased lignin content and wall stiffness.

Specific gene expression patterns have become molecular hallmarks of plant touch response, most notably induction of the *TCH* and similar genes. Orthologs of wall modifying XTHs and calcium binding genes are upregulated in response to mechanical stimulation across species, and as we present here, in *B. distachyon*. Touch elicits major global changes in gene expression, with 2.5% to 10% of the genes assayed in *A. thaliana* and poplar differentially expressed (Lee et al., 2005; Pomiès et al., 2017; Van Moerkercke et al., 2019). In sorghum (*Sorghum bicolor*), leaf sheath imposes mechanical constraints on emerging buds, and removing this dramatically altered gene expression, with 42% of genes differentially expressed over a 9 h period (Liu & Finlayson, 2019). Recent work in cereal species, including oat, wheat, and barley, showed 2-5% differential gene expression within 2 h of mechanostimulation of young leaves with a soft brush (Darwish et al., 2023). In our analysis, we applied a *q*-value significance cutoff of 0.1, which accounts to some extent for the larger number of genes we find differentially expressed compared to other reports. An interactive application (https://hazenlab.shinyapps.io/swiztc) allows users to sort gene expression results by *q*-value, *p*-value, or log-2 fold-change, and may be used by readers to make their own comparisons. Our results are similar to studies in other species in the strong induction of canonical TCH genes, along with stress-related genes (Pomiès et al., 2017). Canonical touch-responsive genes such as the orthologs of *TCH1-4* and related *XTH* and *CML* genes were upregulated immediately following stimulus in wildtype. We also note the previously unreported touch-induction of GH17 and GH18 family genes, as well as *CSLF6*, suggesting a role for β−1,3 glucan modification and MLG and in grass touch response. Furthermore, the pronounced enrichment of chitinases within GH18 may imply their involvement in biotic stress response pathways. Fungal hyphal invasion inherently entails a mechanical aspect, potentially triggering both chitinases for degradation and callose modifiers for cell wall patching in response.

Touch-regulated expression of secondary cell wall-related transcripts appears to differ between early and late timepoints. Upon touch, we observed immediate repression of many key cell wall biosynthetic enzymes, followed by significant upregulation 1 h after stimulus. In poplar, no significant repression of these transcripts was reported, but terms related to wood or cell wall synthesis were also not enriched among touch-regulated transcripts until 24 or 72 h after touch (Pomiès et al., 2017). In other cereals, genes related to cell wall polysaccharides were upregulated 25-60 min following touch while lignin biosynthetic genes down regulated (Darwish et al., 2023). While differences in mechanostimulation, tissue type, gene expression quantification, and temporal sampling schemes complicate direct comparisons of plant touch response experiments, together these data suggest that delayed induction of secondary cell wall transcripts is a common feature of plant touch responses.

The transcriptome response in *SWIZ-OE* before and following touch treatment is notable in that *SWIZ-OE* at 0 min consistently clustered most closely with wildtype at 10 min, indicating that the transcriptome of an untouched *SWIZ-OE* root is similar to an early-touch response wildtype. This is likely due to the presence of some SWIZ protein in the nucleus before touch. Following touch, the remaining cytoplasmic SWIZ protein translocates to the nucleus, and in some gene clusters we observe downregulation of genes in *SWIZ-OE* that were upregulated in wildtype. This inversion may suggest several possible mechanisms, such as SWIZ having a repressive function, or SWIZ activating a transcription circuit that triggers quick repression of touch-induced genes.

The timing of SWIZ nuclear accumulation following mechanostimulation was consistent with *A. thaliana* Group I bZIPs (Tsugama et al., 2014, 2016). However, by adopting a finer temporal sampling scheme, and quantifying nuclear signal sooner after touch (2 vs. 30 min), our results clarify the rapidity of this translocation. Furthermore, we show that the speed and magnitude of SWIZ nuclear accumulation is not diminished over successive touch treatments. Poplar transcriptional response to a second touch stimulus was significantly attenuated relative to a single treatment and trees given daily touch treatment for 4 days no longer showed touch-induced increases in their rates of radial growth (Martin et al., 2010; Pomiès et al., 2017). If *B. distachyon* is likewise desensitised to repeated touch, the repeatability of SWIZ translocation implies a mechanism downstream of touch perception and bZIP translocation in mediating that shift.

Although we did not observe a robust secondary wall response in touched stems, touch-responsive wall synthesis is still implicated by our transcriptomic data. Two CREs not previously associated with touch response, the AC and VNS elements, were identified in our touch-responsive and SWIZ-targeted datasets. These sites are bound by NAC and MYB proteins that regulate the thickening of secondary cell walls (Handakumbura et al., 2018; Kim et al., 2012; Ohtani et al., 2011; Olins et al., 2018; Tamura et al., 2019; Zhong et al., 2011; Zhong & Ye, 2012).

Before touch, several genes in the monolignol biosynthetic pathway were significantly downregulated in *SWIZ-OE* plants. Following touch, most of these genes were significantly upregulated. This activity is consistent with the compositional data presented by Gladala-Kostarz et al. (2020). This activation may be a result of direct binding of SWIZ, or an indirect effect from other transcriptional regulators like the NAC transcription factor *SWN5* that is significantly upregulated in *SWIZ-OE* following touch and capable of activating the full developmental programme for secondary cell wall synthesis (Valdivia et al., 2013). The E-box (CANNTG) was initially described as a bHLH binding motif; many bZIP phylogenetic groups in *A. thaliana* have been shown to bind similar sequences (O’Malley et al., 2016). Using the same technique, we identified the SWIZ binding motifs as (A/C)CAGNCTG(T/G) and (A/C)CAGCTG(T/G), very similar to those bound by *A. thaliana* orthologs, although an ambiguous nucleotide in the core of one variant is not reported for other Group I bZIPs. Genes bound by SWIZ *in vitro* show both activation and repression following touch, suggesting a complex regulatory function for SWIZ. Furthermore, SWIZ direct binding sites were found in both promoter regions and gene bodies, and most often in genes activated by touch. SWIZ gene body binding targets tended to be repressed in *SWIZ-OE* plants following touch, without either being clearly associated with up- or down-regulation. AtVIP1 and AtbZIP29 have both been described as activators (Pitzschke et al., 2009; Ringli & Keller, 1998; Van Leene et al., 2016; Yin et al., 1997), while AtbZIP18 is described as a repressor (Gibalová et al., 2017; Tsugama et al., 2012). The directionality of SWIZ transcriptional control remains to be determined.

## 4. Conclusions

Touch significantly remodelled the *B. distachyon* transcriptome, with notable changes in wall polysaccharide biosynthetic gene expression not previously reported and revealed an enrichment of secondary cell wall-associated CREs, the AC and VNS elements. SWIZ translocation dynamics are similar to other bZIP proteins, and the timing of this translocation is consistent with the differential touch-responsive gene expression in *SWIZ-OE*. Some of the data presented here rely on overexpression of *SWIZ*, which comes with certain limitations as to how directly the phenotypic and molecular results relate to SWIZ-specific function in wildtype plants. Further experimentation is needed to elucidate the bZIP regulatory network in response to touch and other stimuli.

## 5. Materials and methods

### 5.1. Phylogenetic analysis

Protein sequences described for *A. thaliana*, *B. distachyon* and *O. sativa* (Liu & Chu, 2015) as Group II bZIPs were selected and searched against the most recent genome annotations in Phytozome v12.1 (https://phytozome.jgi.doe.gov). The *Nicotiana tabacum* homologs NtRSGa and NtRSGb were also added to the analysis. Protein sequences were aligned by MAFFT using the L-INS-I model (Katoh et al., 2019). A maximum-likelihood phylogenetic tree bootstrap resampling value of 1000 was generated in W-IQ-TREE (Trifinopoulos et al., 2016). All proteins in the phylogenetic analysis are described in Supplemental File S1.

### 5.2. Plant transformation

Overexpression constructs and plant transformation were carried out as previously described using accession Bd21-3 (Handakumbura et al., 2013). Bradi1g17700 coding sequence was amplified from cDNA and cloned into the pOL001 ubigate ori1 binary expression vector (Handakumbura et al., 2018) to make the *SWIZ-OE* trangene. The coding sequence without stop codon was amplified and cloned in frame with the enhanced GFP coding sequence in the pOL001 vector to generate *SWIZ:GFP-OE*. For both constructs, three independent events were analysed with no phenotypic difference between them for height or translocation dynamics among the *SWIZ:GFP-OE* lines.

### 5.3. Translocation assay

Bd21-3 seeds were surface sterilised and grown vertically on 1X MS media, pH 5.7, without sucrose for 6 days at 28°C in the dark. After 6 days, seedlings were moved to treatment plates containing 1X MS media, pH 5.7.

All observations were made on a Nikon A1R scanning confocal microscope using a Plan Apo 10× 0.5NA objective and PMT detector. Root areas were located by eye using transmitted light and then imaged with excitation at 488 nm and emission captured at 510–530 nm. Roots were imaged for 30 min pre-treatment, with images captured every 2 min. The one exception is the top panel of Figure 1b, which was made from observations on an Olympus FV3000 point scanning confocal laser microscope with a 10×/NA 0.4 objective and HyD detectors, with excitation at 488 nm and emission collection at 510–530 nm.

To elicit the touch response, the observed root region was gently probed five times in ~5 sec with a blunt probe while observing through the eyepiece (Supplemental Figure S7). Images were captured for 60–90 min post-treatment. For experiments with multiple stimulus events, the timelapse sequence was paused and roots were probed as described for the relevant stimulus events.

Analysis of GFP signal was done using the Nikon NIS Elements Advanced Research V5 software package. Nuclear regions were thresholded for intensity and particle size to identify regions of interest. Fluorescence intensity was calculated for each ROI and averaged at each timepoint. For the top panel of Figure 1b, analysis was carried out in ImageJ with thresholding for intensity and particle size to identify regions of interest.

### 5.4. Thigmomatic construction and operation

The Thigmomatic is a basic robotic device that sweeps plants with a metal bar at regular intervals to elicit a touch response. The device was constructed from aluminium V-Slot linear rail (Openbuilders Partstore, Monroeville, NJ) and bracket joints for the upright supports (20 mm × 20 mm), cross bars (20 mm × 20 mm), and tracks (20 mm × 40 mm). Two gantry carts ride along the 20 mm × 40 mm V-Slot linear rails, connected by a 6.35 mm diameter metal rod bolted to the carts. Their movement is powered by a belt-driven linear actuator system using a NEMA 17 stepper motor with a 12V 18W AC/DC power supply. Motor function is controlled by a Raspberry Pi 3B microcomputer with stepper motor HAT (Adafruit Industries, New York). The Thigmomatic was programmed to cover a specified distance in one direction once every 90 min.

### 5.5. Transverse stem sections and histology

Internode segments of the main stem were embedded in 8% agarose. A Leica VT1000 Vibratome was used to make 55 μm thick transverse sections. Histochemical staining with phloroglucinol-HCl was done as previously described (Matos et al., 2013). Images were obtained at 4, 10 and 20X using a Nikon Eclipse E200MV R light microscope and PixeLINK 3 MP camera. Cell wall thickness was quantified for interfascicular fibre cells separated by one cell layer from the mestome cells on the phloem side of major vascular bundles. Using ImageJ, lines were drawn across two adjoining walls divided by two yielding one cell wall width.

### 5.6. RNA extraction and quantification and analysis

Seedlings were grown for 6 days on vertical 1X MS agar plates. Touch treatment was performed as described above using a metal probe along the entire length of the root. Untouched samples were collected immediately before touch treatment and touched samples were collected 10, 30, and 60 min post-treatment. Three roots were pooled per biological replicate, and RNA was extracted by Qiagen RNeasy Plant Mini Kit with on-column DNA digestion with RNase-free DNase I (Qiagen). Strand-specific libraries were prepared using the Illumina TruSeq kit. Libraries were sequenced using Illumina technology and processed as previously described (MacKinnon et al., 2020). Briefly, quality was checked with FastQC (Andrews, 2010), aligned to the Bd21 reference genome (v3.1) using HiSat2 (Kim et al., 2015), then assembled and quantified using StringTie (Pertea et al., 2015). Transcripts were normalised and differential expression tested using the likelihood ratio test from the R (v3.6.0) package DESeq2 (Love et al., 2014). Benjamini-Hochberg *p*-value adjustments were applied to account for multiple testing with a significance cutoff of 0.1 and 30,380 of 34,310 reference genes had non-zero read counts after normalisation, with an average mapping percentage of 97.8% for all libraries, as determined by SAMtools (Li et al., 2009). Heatmaps were generated using median gene expression scaled by row, with zero-expression genes removed. Hierarchical clustering was performed using Euclidean distances with complete clustering and k-means grouping. Specific treatment contrasts (i.e., wildtype vs *SWIZ-OE*) were compared by Wald test from DESeq2 (Love et al., 2014). Statistical enrichment of gene families was assessed using Fisher’s exact test. Raw read data were deposited in the European Nucleotide Archive for public access (Accession no.: E-MTAB-10084).

### 5.7. DNA affinity purification sequencing

DNA affinity purification was carried out as previously described (Handakumbura et al., 2018). In brief, transcription factor coding sequences were HALO tagged and mixed with Bd21 genomic DNA for *in vitro* binding. Protein-DNA was crosslinked, fragmented, immunoprecipitated using the HALO antibody, barcoded, and sequenced. Reads were mapped to the Bd21 genome using HiSat2 (Kim et al., 2015) to identify binding target loci. Peak calling and motif analysis were done using HOMER v4.10 (Hypergeometric Optimization of Motif EnRichment) suite (Heinz et al., 2010). Motif enrichment was calculated against the hypergeometric distribution; the significance threshold was set to *p* < 0.05. The nearest annotated gene to a bound peak was used for GO analysis. Raw read data were deposited in the European Nucleotide Archive for public access (Accession no.: E-MTAB-10066).

### 5.8. Gene ontology analysis

Phytozome was used to find orthologs for all *B. distachyon* v3.1 genes as the reciprocal best match to *A. thaliana* TAIRv10 protein sequences. Arabidopsis gene identifiers were submitted to g:Profiler (Raudvere et al., 2019) for KEGG and Wiki pathway enrichment analysis.

### 5.9. iDREM network analysis

Probabilistic graphical models that predict diverging gene expression paths were generated using iDREM (Ding et al., 2018). Briefly, this software applies an input-output hidden Markov model to time course gene expression data overlaid with static regulatory information, in this case SWIZ DAP-seq protein-DNA interactions. GO analysis, described above, was performed for each path identified by iDREM.

### 5.10. *Cis*-*regulatory sequence analysis*

Differentially expressed genes following touch were categorised as increasing or decreasing in transcript abundance at each time point. Homer v.4.10 identified regulatory sequences in the 1000 bp upstream of the transcriptional start site of differentially expressed genes previously identified in *A. thaliana* DAP-seq analysis (Heinz et al., 2010; O’Malley et al., 2016). We also applied the growing k-mer approach to identify CREs (Moore et al., 2022). In brief, touch-responsive genes were divided into six groups: up or down regulated each timepoint. The 1000 bp upstream of differential expressed genes were searched for all possible 6-mers, plus one additional base (A, T, C and G), and then tested for *p*-value shift. If the *p*-value was lower, the 7-mer(s) was kept and grown further up to 12-mer length. The Tomtom tool in MEME Suite 5.4.1 found similarities between significant CREs and the *A. thaliana* DAP-seq database, with a false discovery rate cutoff of < 0.01.

### 5.11. Root touch experiment

Bd21-3 seeds were surface sterilised and plated on 1× MS, pH 5.7, containing 0.05 % MES as a buffering agent and 1% plant agar (Gold Bio). Seeds were stratified on plates in the dark at 4 °C for 2 days and then transferred to a Percival PGC-15 growth chamber with day/night conditions of 16 h light at 24 °C and 8 h dark at 18 °C, respectively, and grown at a ~10° angle from vertical. After 2 days, a touch location was designated by selecting the lower of 1 cm up from the root tip or 1 cm down from the seed and marked on the plate. For 5 days, this marked spot was treated twice daily, 2 h before and 2 h after chamber midday (ZT6 and ZT10). Touch treatment consisted of five firm presses with the side of a sterile pipette tip.

### 5.12. Root length and straightness measurement

Plates were photographed at the conclusion of the experiment. Semi-automated measurements of root length were performed using the Smart Roots plugin for ImageJ (Lobet et al., 2011). Straightness was quantified as described in Swanson et al. (2015); the straight-line distance between the root tip and the base of the seed was measured, and this value was divided by the traced length of the root.

## Supporting information

Coomey et al. supplementary material Coomey et al. supplementary material

## Data Availability

The data that support the findings of this study are openly available at E-MTAB-10066 and E-MTAB-10084 in the European Nucleotide Archive and https://hazenlab.shinyapps.io/swiztc.

## Accession number

AtbZIP18 (At2g40620), AtbZIP29 (At4g38900), AtbZIP52 (At1g06850), AtTCH1/AtCaM2 (At2g41110), AtTCH2/AtCML24 (At5g37770), AtTCH3/CML12 (At2g41100), AtTCH4/AtXTH22 (At5g57560), CAD1 (Bradi3g17920), CESA4 (Bradi4g28350), COMT6 (Bradi3g16530), CSLF6 (Bradi3g16307), GA2ox3 (Bradi2g50280), SWAM3/MYB44 (Bradi1g30252), NAC35 (Bradi2g28020), NtRSGa (Niben101Scf01150g00005), NtRSGb (Niben101Scf02191g00014), SWIZ (Bradi1g17700) and VIP1 (At1g43700).

## Supplementary materials

The supplementary material for this article can be found at http://doi.org/10.1017/qpb.2024.5.
