# Supplementary material for: Mechanically induced localisation of SECONDARY WALL INTERACTING bZIP is associated with thigmomorphogenic and secondary cell wall gene expression
Source: Quant Plant Biol. 2024 May 3;5:e5. doi: 10.1017/qpb.2024.5 (PMC11106548; doi:10.1017/qpb.2024.5)
Supplement: Coomey et al. supplementary material [file S2632882824000055sup001.docx]

**
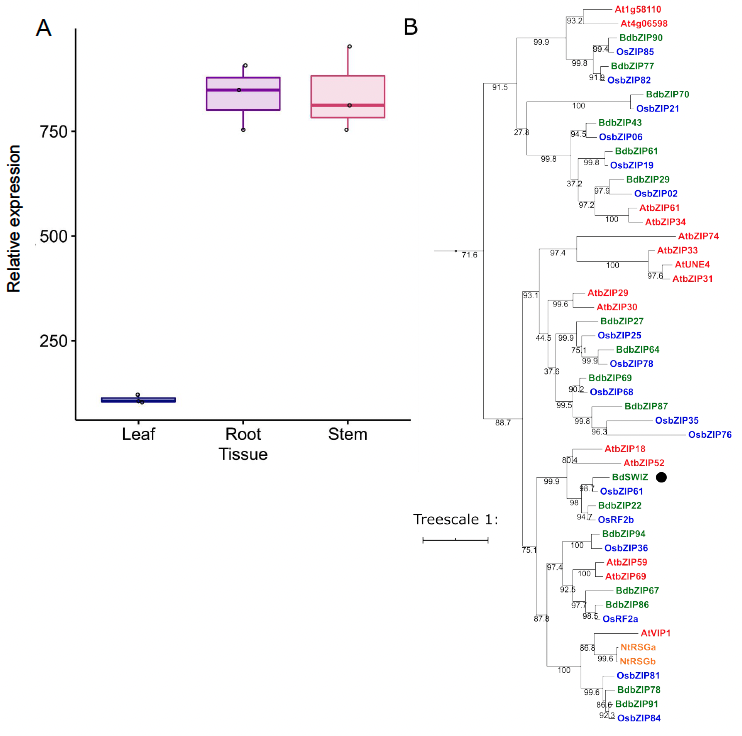
 Supplemental Figure S1. SWIZ is a Group I bZIP highly expressed in maturing stem and root.** (A) *SWIZ* transcript abundance in *Brachypodium distachyon* leaf, root, and stem tissue measured by microarray. Mean +/- standard deviation of three biological replicates. (B) Phylogeny analysis of amino acid sequences from *B. distachyon* (green), *Oryza sativa* (blue), *Arabidopsis thaliana* (red), *Nicotiana tabacum* (orange) shows *SWIZ* (black dot). The phylogeny was reconstructed using maximum likelihood analysis with a bootstrap resampling value of 1000. The numbers labeled on the branches are the posterior probability to support each clade. The protein sequences, full locus IDs, and plant species with genome source are provided in Supplemental File S1.

**
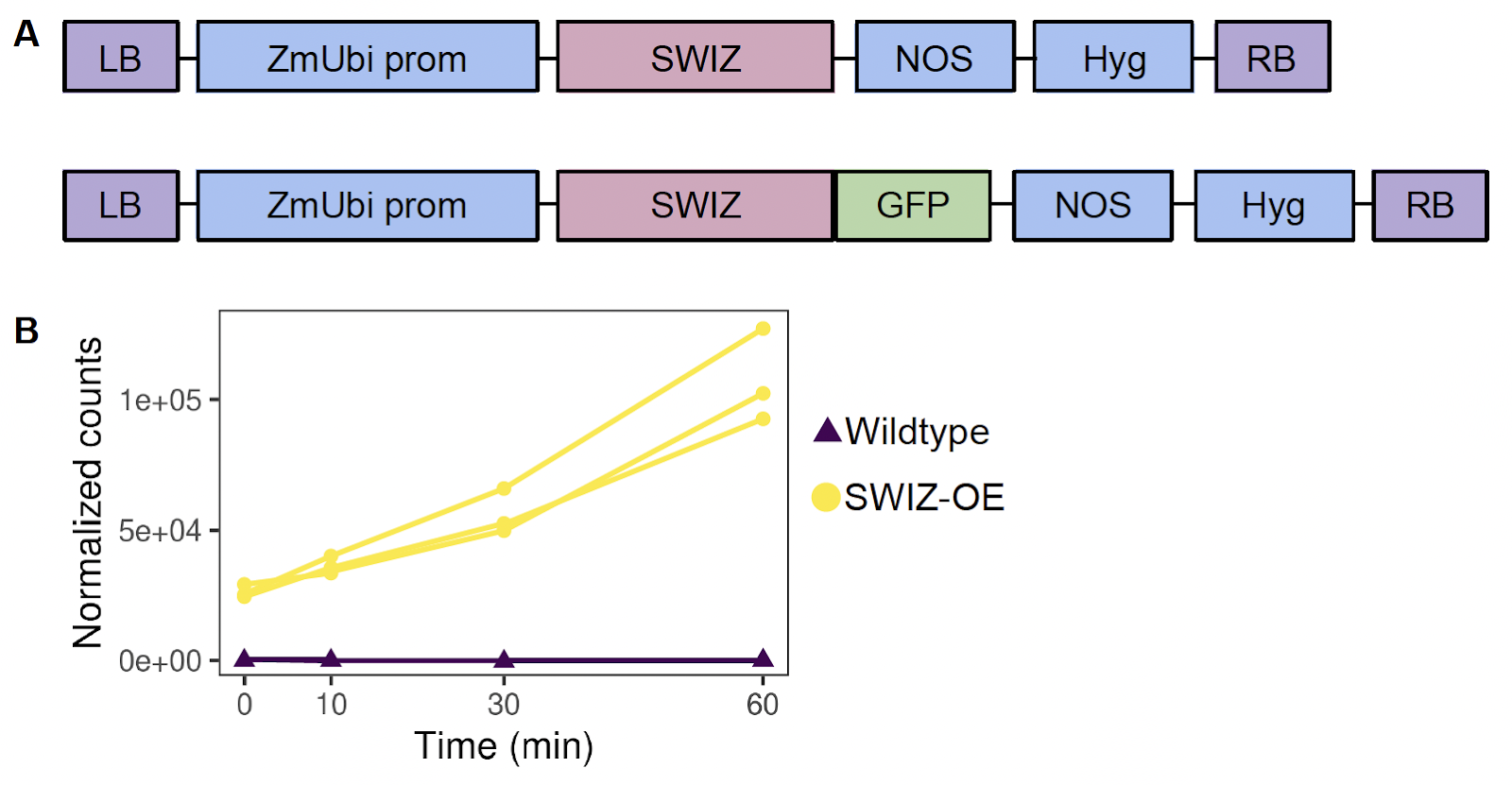
**

**Supplemental Figure S2. Description of *SWIZ* transgenic reagents.** (A) Diagram of *SWIZ* overexpression transgenes. The *ZmUbi* promoter was used to drive expression of the *SWIZ* coding sequence, either alone or fused in frame with *eGFP*. (B) Transcript abundance of *SWIZ* measured by RNA-sequencing. Root tissue was sampled just prior to touch (t = 0), and at 10, 30, and 60 min following touch treatment in wildtype and *SWIZ-OE.* LB, left border; *ZmUbi* prom, maize ubiquitin promoter; Hyg, hygromycin phosphotransferase gene; NOS, nopaline synthase terminator; RB, right border.


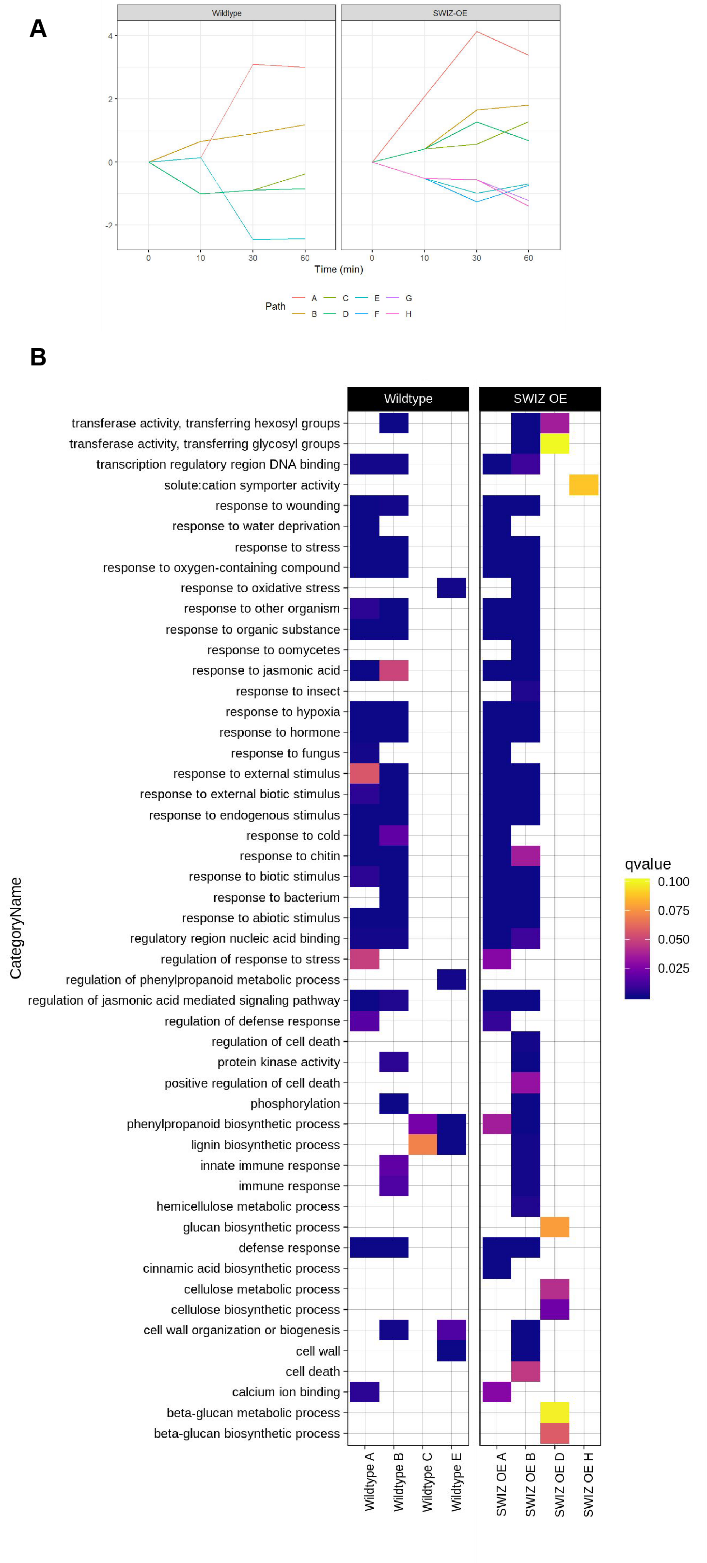


**Supplemental Figure S3. Network analysis of differential gene expression paths and enriched GO terms.** (A) Path determinations from iDREM time course analysis. Each line represents a set of genes with similar expression level patterns over the time course relative to time 0, pre-touch. (B) Heatmap of selected GO term enrichment of genes from iDREM path analysis. Color indicates *q*-value score, with a cutoff of 0.1. Terms without a colored block are not significantly enriched in that path.


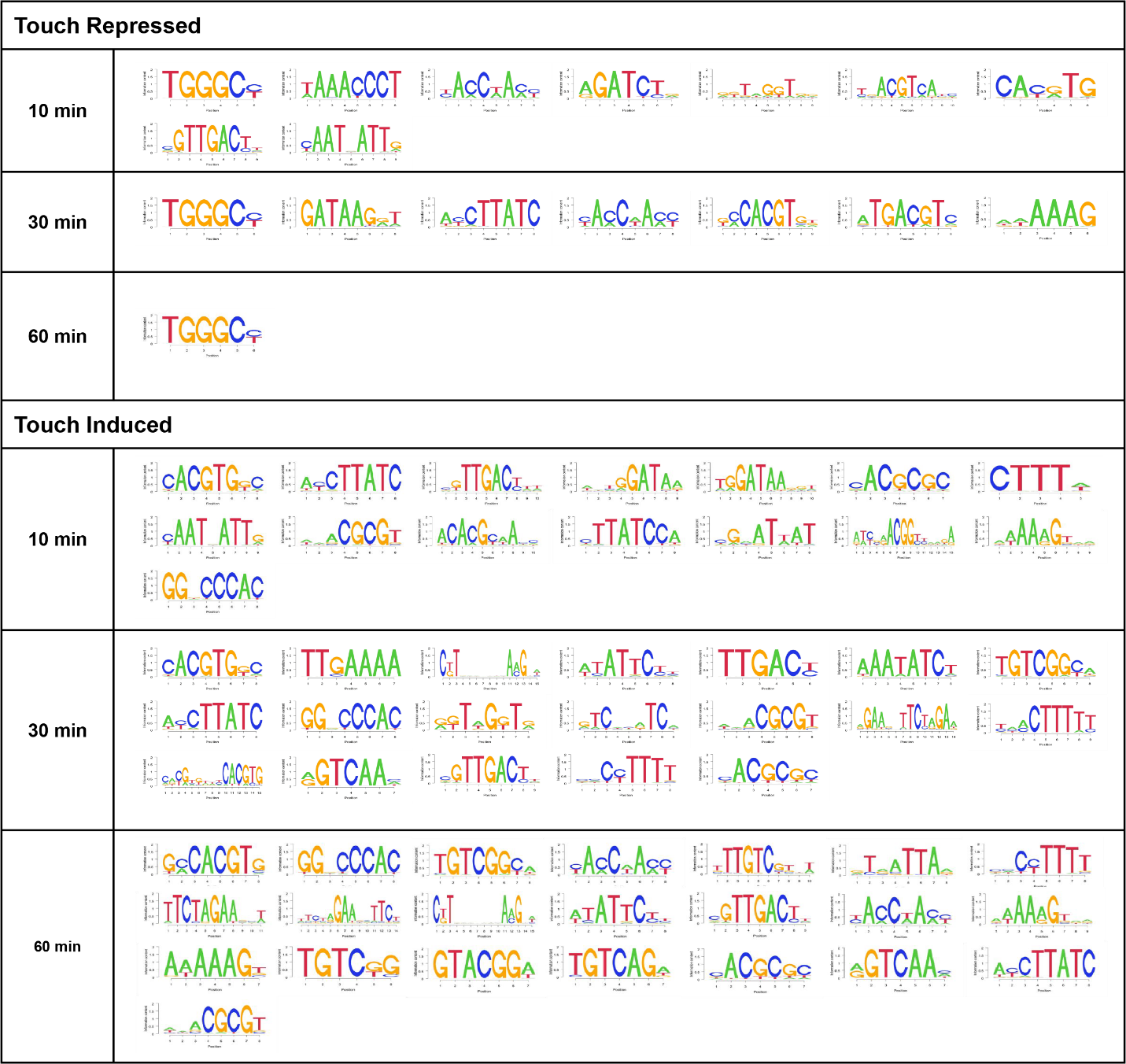


**Supplemental Figure S4. Brachypodium distachyon cis-regulatory sequences most overrepresented at each time course after touching.** Each nucleotide sequence is a position probability matrix motif derived from DNA-affinity purification sequencing and identified as enriched under different time courses. The height of the letter at each position is proportional to the probability of a given nucleotide.


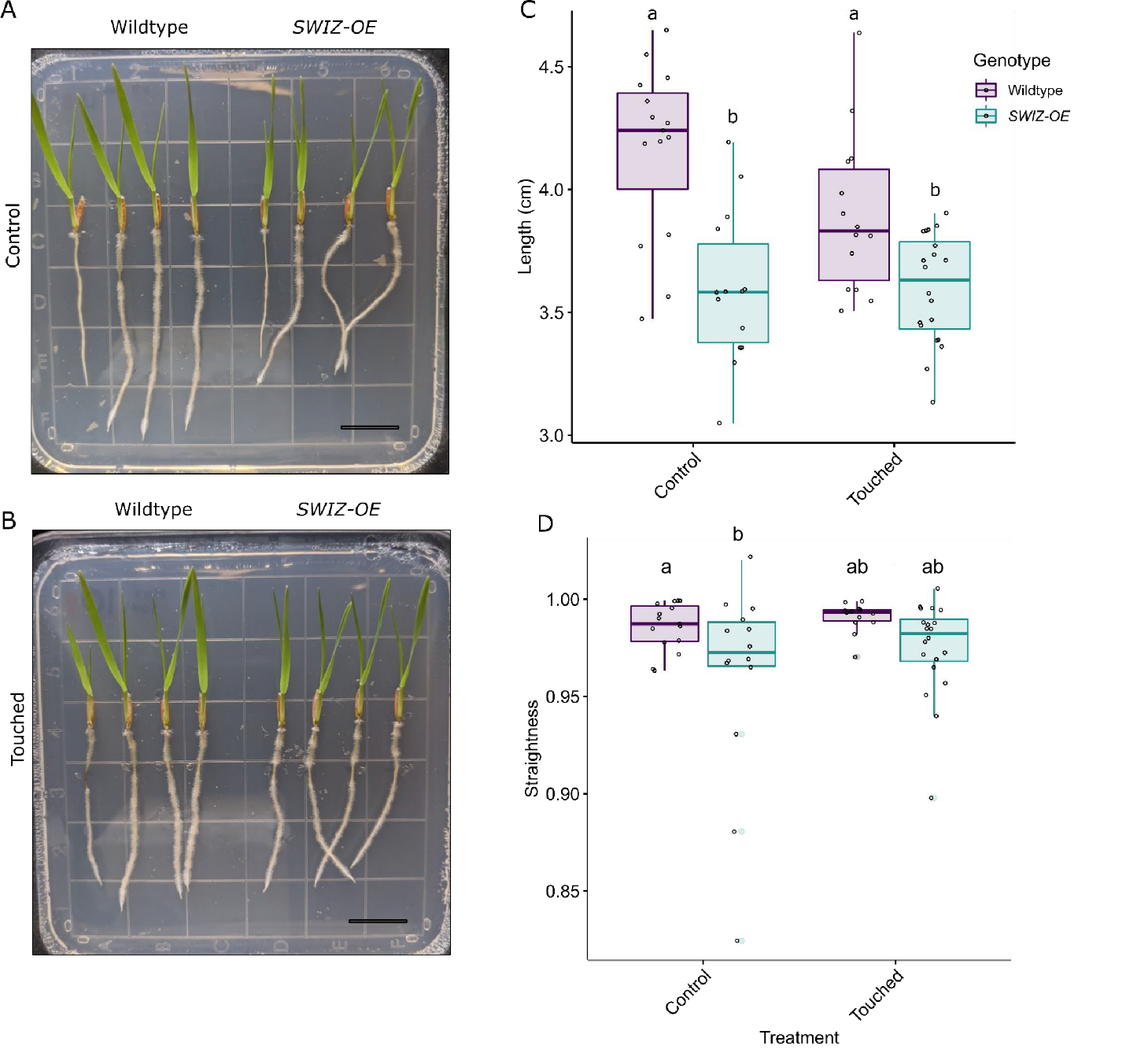


**Supplemental Figure S5. *SWIZ-OE* roots are shorter than wildtype with no significant difference in straightness in response to direct touch.** Wildtype and *SWIZ-OE* seedlings grown on plates under control (A) or touched (B) conditions for five days. Touched plants were probed with a pipette tip twice a day. (C) Quantification of root length in control and touched conditions. Significance denoted by compact letter display reflecting Tukey HSD adjusted *p-*values < 0.05. (D) Quantification of root straightness in control and touch conditions. Significance denoted by * reflecting Wilcoxon sign-ranked test for non-parametric data with *p*-value < 0.05, ns = not significant. Scale bar = 1 cm.


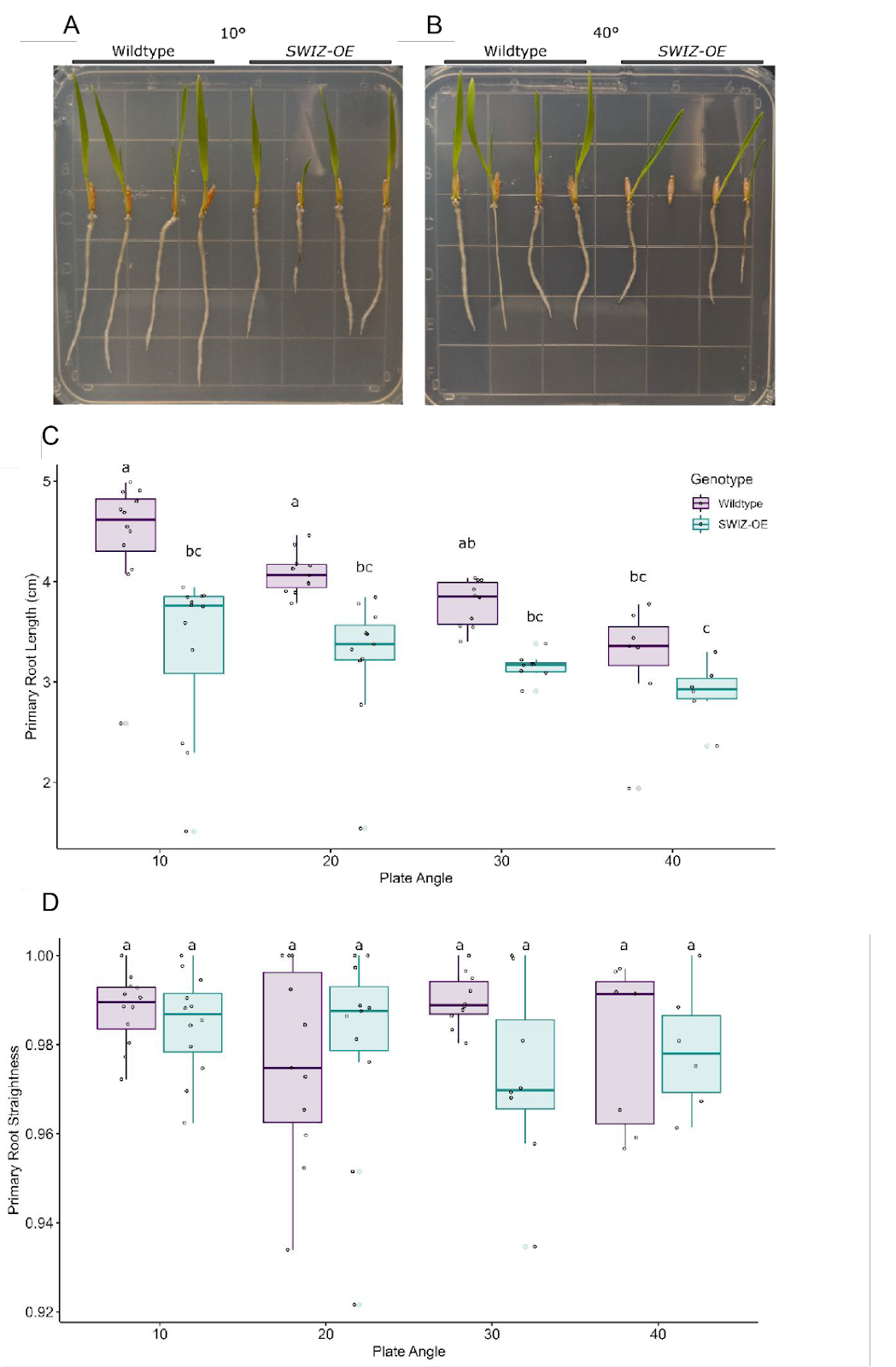


**Supplemental Figure S6. Wildtype roots shorten with increasing plate angle, while *SWIZ-OE* roots are consistently short, with no significant difference in straightness.** Wildtype and *SWIZ-OE* seedlings grown on plates at (A) 10°, 20°, 30°, and (B) 40° incline from vertical. (C) Quantification of root length. (D) Quantification of primary root straightness. Significance denoted by compact letter display reflecting Tukey HSD adjusted *p-*values < 0.05. Scale bar = 1 cm.


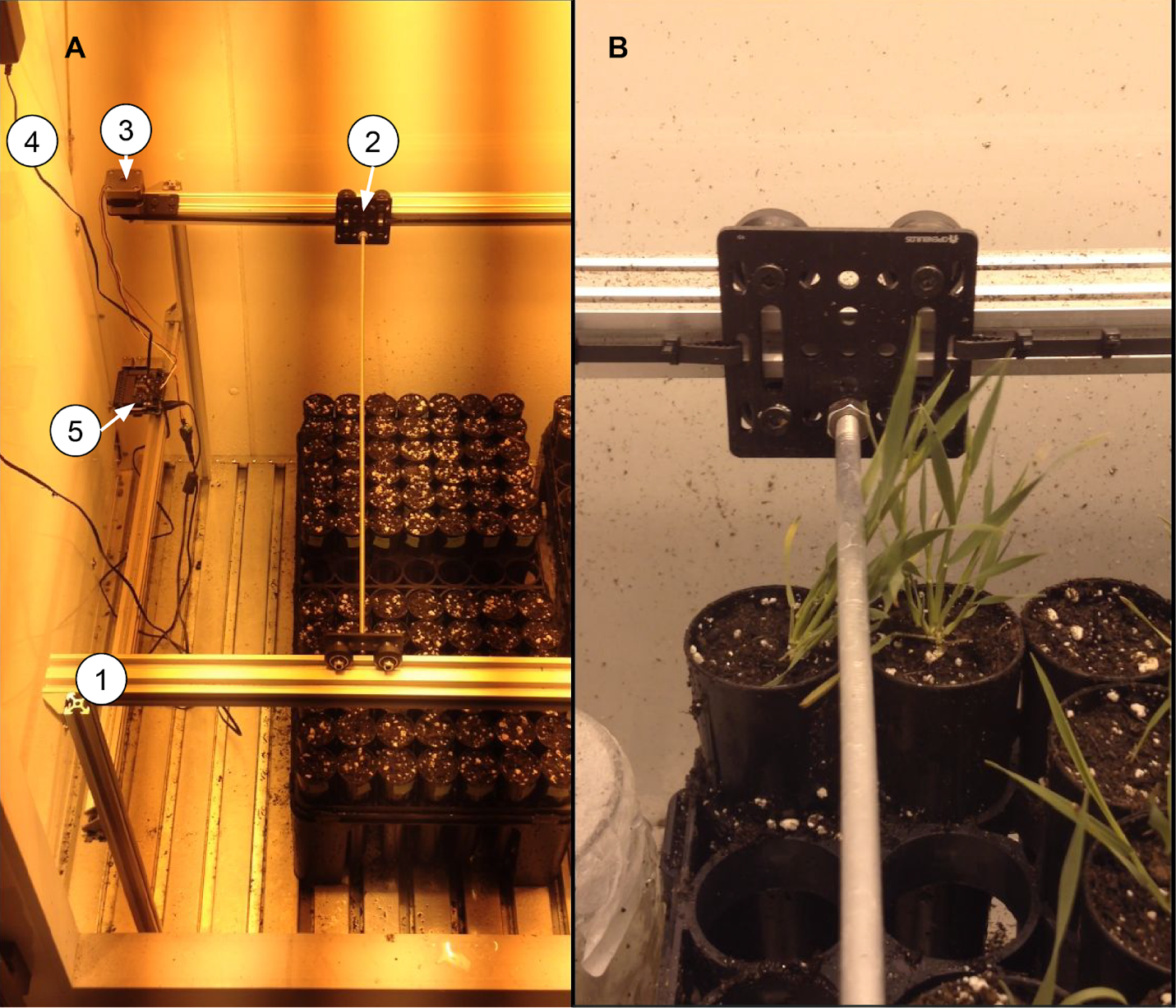


**Supplemental Figure S7. Thigmomatic, an automated system to administer touch stimulus.** (A) Overview of Thigmomatic inside a Percival PGC-15 growth chamber showing linear rail based frame (1), gantry carts (2), NEMA17 stepper motor (3), 12V 18W AC/DC power supply (4), and Raspberry Pi 3b microcomputer (5). (B) Thigmomatic making contact with a *Brachypodium distachyon* plant.

**
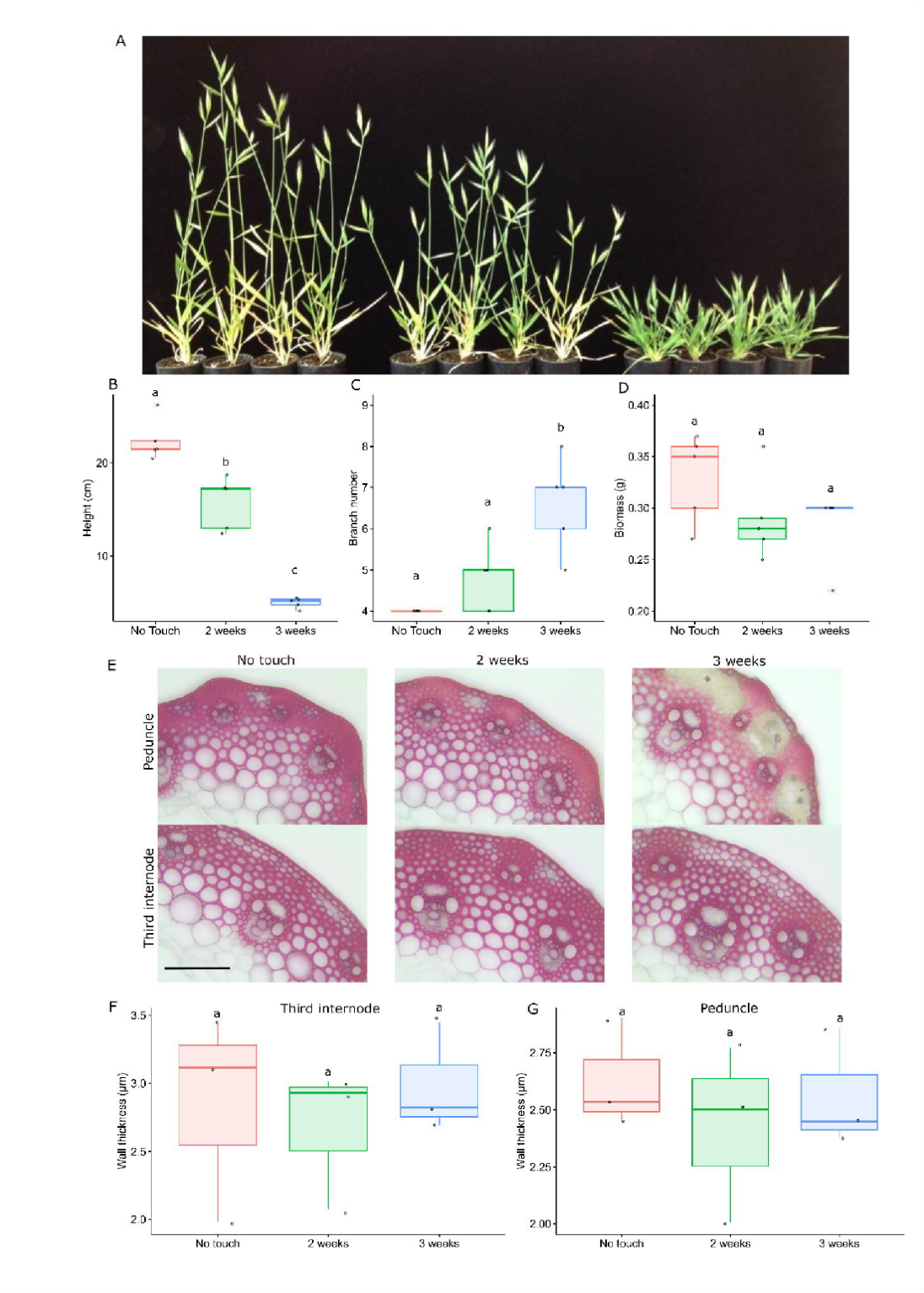
**

**Supplemental Figure S8. Brachypodium distachyon displays classic thigmomorphogenic phenotypes.** One-week-old wildtype plants were treated with touch stimulus every 90 min. (A) Left to right, plants that experienced no stress, two weeks stress, three weeks stress and were then allowed to recover, imaged one week after the end of treatment (B) height, (C) aboveground non-grain biomass weight, and (D) branch number were measured at senescence. n = 4-5 plants per treatment. Significance denoted by compact letter display reflecting Tukey HSD adjusted *p*-values < 0.05. (E) Transverse sections of the peduncle or third internode were taken from control, 2 week stressed, and 3 week stressed plants and stained with phloroglucinol-HCl. (F) Quantification of interfascicular fiber wall thickness. Scale bar = 100 µm. n = 3 plants per treatment.

**
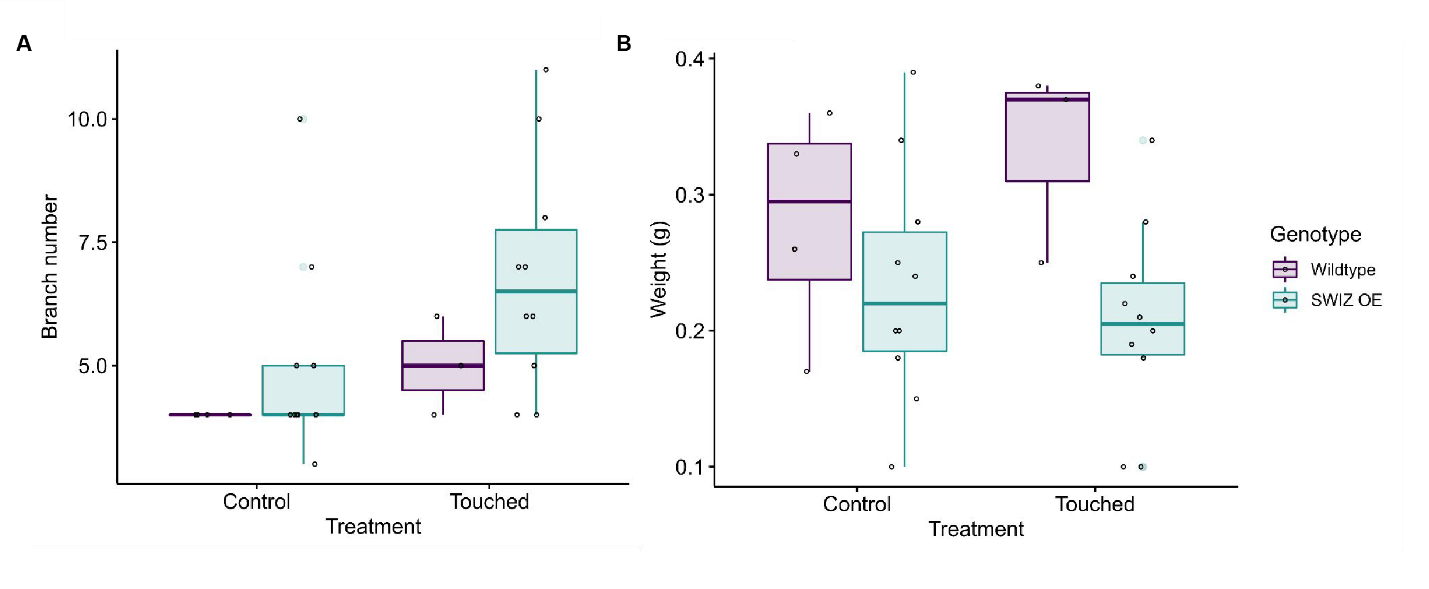
**

**Supplemental Figure S9. Biomass and branching in *SWIZ-OE* under touch treatment shows no difference from wildtype.** Wildtype and *SWIZ-OE* plants were placed under control conditions or received two weeks of mechanical stress every 90 min. After senescence, branch number (A) and aboveground biomass weight (B) were quantified. Significant differences were not observed following ANOVA and Tukey HSD testing.

**
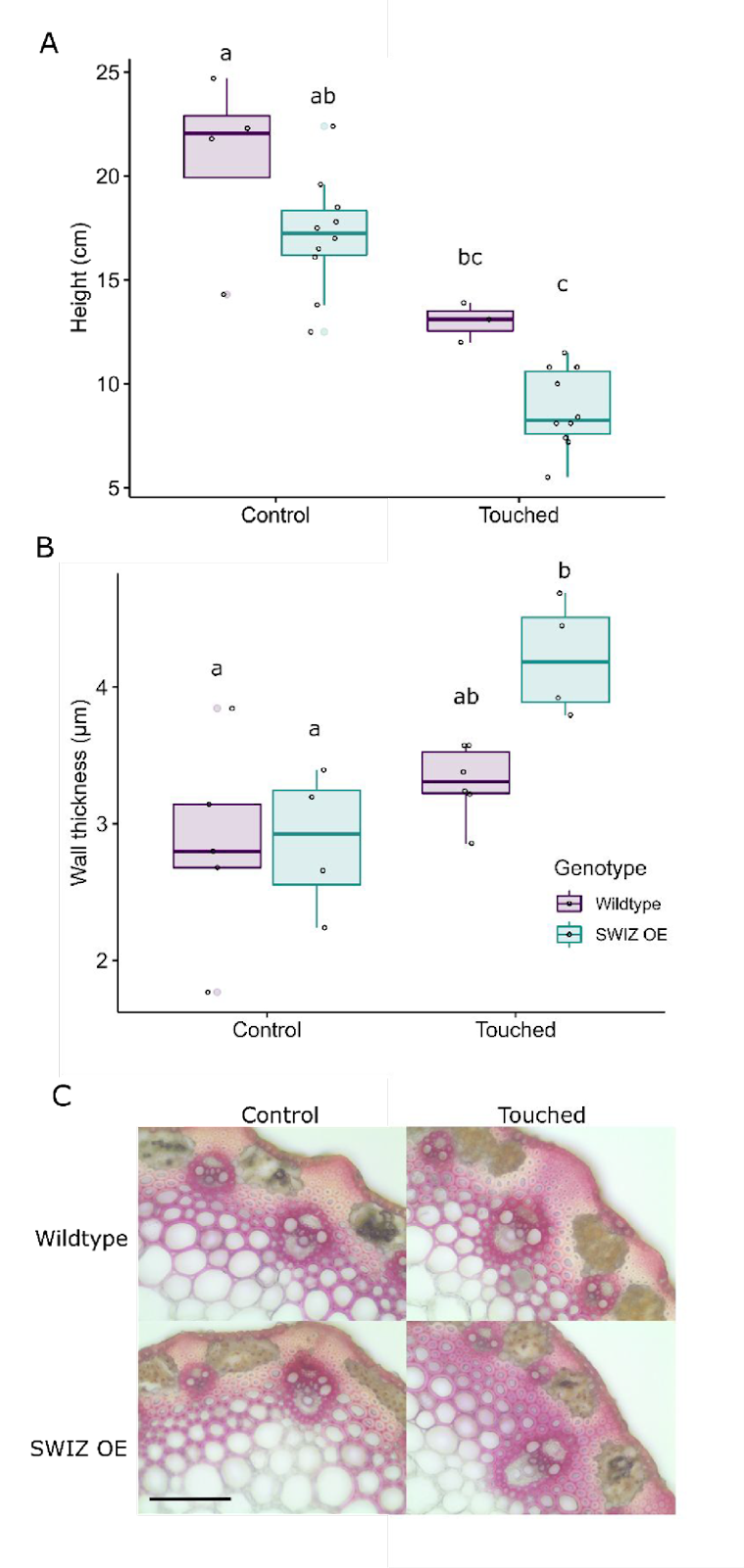
**

**Supplemental Figure S10. *SWIZ-OE* marginally enhanced thigmomorphogenic response in stems.** Wildtype and *SWIZ-OE* plants were placed under control conditions or received two weeks of mechanical stress every 90 min. (A) Quantification of main stem height at senescence. (B) Quantification of interfascicular fiber wall thickness in transverse cross sections of the peduncle. n = 4 to 6 plants per genotype, per treatment. (C) Representative cross sections stained with phloroglucinol-HCl. Scale bar = 100 µm. Significance denoted by compact letter display reflecting Tukey HSD adjusted *p-*values < 0.05.

**
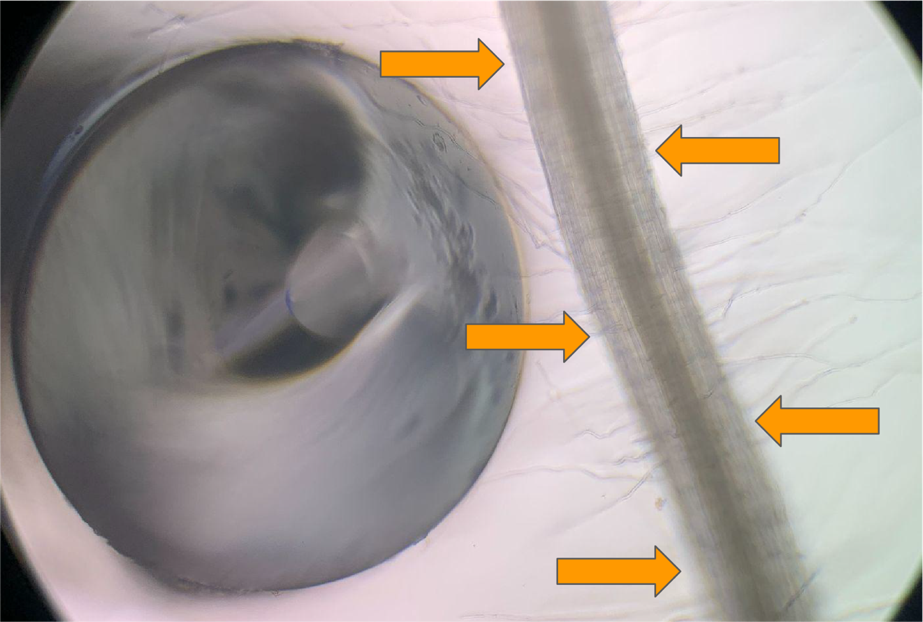
**

**Supplemental Figure S11. Method of root touch treatment.** A blunt probe formed from a glass Pasteur pipette was used to gently tap on the root five times in areas like those indicated by the orange arrows.
