# Supplementary material for: Mechanically induced localisation of SECONDARY WALL INTERACTING bZIP is associated with thigmomorphogenic and secondary cell wall gene expression
Source: Quant Plant Biol. 2024 May 3;5:e5. doi: 10.1017/qpb.2024.5 (PMC11106548; doi:10.1017/qpb.2024.5)
Supplement: Peer review history

# Editor report: Recommendation: Mechanically induced localisation of SECONDARY WALL INTERACTING bZIP is associated with thigmomorphogenic and secondary cell wall gene expression — R0/PR2

**DOI:** 10.1017/qpb.2024.5.pr2

**Affiliations:** RDP, ENS de Lyon, France

Dear Samuel,

We now have received two constructive reviews for your article. There is certainly interest, and the story perfectly fits the scope of the journal. Yet, the reviewers also raise a number of important points to address before acceptance. Please take all of them into account for the revision.

Best wishes,

Olivier

# Editor report: Decision: Mechanically induced localisation of SECONDARY WALL INTERACTING bZIP is associated with thigmomorphogenic and secondary cell wall gene expression — R0/PR3

**DOI:** 10.1017/qpb.2024.5.pr3

**Affiliations:** RDP, ENS de Lyon, France

No accompanying comment.

# Editor report: Recommendation: Mechanically induced localisation of SECONDARY WALL INTERACTING bZIP is associated with thigmomorphogenic and secondary cell wall gene expression — R1/PR5

**DOI:** 10.1017/qpb.2024.5.pr5

**Affiliations:** RDP, ENS de Lyon, France

As you can see the reviewers are quite polarized. Reviewer 2 has noted a number of shortcomings, but in my view, these can be relatively easily fixed with text/figure changes and toning down (once checked that the results are indeed correct). I therefore invite the authors to revise their manuscript, taking into account this careful analysis from reviewer 2.

# Editor report: Decision: Mechanically induced localisation of SECONDARY WALL INTERACTING bZIP is associated with thigmomorphogenic and secondary cell wall gene expression — R1/PR6

**DOI:** 10.1017/qpb.2024.5.pr6

**Affiliations:** RDP, ENS de Lyon, France

No accompanying comment.

# Editor report: Recommendation: Mechanically induced localisation of SECONDARY WALL INTERACTING bZIP is associated with thigmomorphogenic and secondary cell wall gene expression — R2/PR8

**DOI:** 10.1017/qpb.2024.5.pr8

**Affiliations:** RDP, ENS de Lyon, France

As you can see, the reviewer and myself are happy with the revised version of the manuscript - Congratulations!

# Editor report: Decision: Mechanically induced localisation of SECONDARY WALL INTERACTING bZIP is associated with thigmomorphogenic and secondary cell wall gene expression — R2/PR9

**DOI:** 10.1017/qpb.2024.5.pr9

**Affiliations:** RDP, ENS de Lyon, France

No accompanying comment.
